# Quantification of Neurite Degeneration with Enhanced Accuracy and Efficiency in an *In Vitro* Model of Parkinson’s Disease

**DOI:** 10.1523/ENEURO.0327-21.2022

**Published:** 2022-03-17

**Authors:** Rachel T. Clements, Lauren E. Fuller, Kyle R. Kraemer, Samantha A. Radomski, Sarah Hunter-Chang, Wesley C. Hall, Alborz A. Kalantar, Bradley R. Kraemer

**Affiliations:** 1 Department of Biological Sciences, Eastern Kentucky University, Richmond, KY 40475; 2 Department of Psychology, Birmingham-Southern College, Birmingham, AL 35254; 3 Neuroscience Graduate Program, University of Virginia, Charlottesville, VA 22903

**Keywords:** axonal degeneration, degeneration index, neurite degeneration, neurodegeneration, oxidative stress, Parkinson’s disease

## Abstract

Neurite degeneration is associated with early stages of neurodegenerative disorders such as Alzheimer’s disease, Parkinson’s disease (PD), and amyotrophic lateral sclerosis. One method that is commonly used to analyze neurite degeneration involves calculation of a Degeneration Index (DI) following utilization of the Analyze Particles tool of ImageJ to detect neurite fragments in micrographs of cultured cells. However, DI analyses are prone to several types of measurement error, can be time consuming to perform, and are limited in application. Here, we describe an improved method for performing DI analyses. Accuracy of measurements was enhanced through modification of selection criteria for detecting neurite fragments, removal of image artifacts and non-neurite materials from images, and optimization of image contrast. Such enhancements were implemented into an ImageJ macro that enables rapid and fully automated DI analysis of multiple images. The macro features operations for automated removal of cell bodies from micrographs, thus expanding the application of DI analyses to use in experiments involving dissociated cultures. We present experimental findings supporting that, compared with the conventional method, the enhanced analysis method yields measurements with increased accuracy and requires significantly less time to perform. Furthermore, we demonstrate the utility of the method to investigate neurite degeneration in a cell culture model of PD by conducting an experiment revealing the effects of c-Jun N-terminal kinase (JNK) on neurite degeneration induced by oxidative stress in human mesencephalic cells. This improved analysis method may be used to gain novel insight into factors underlying neurite degeneration and the progression of neurodegenerative disorders.

## Significance Statement

Neurite degeneration is a cellular event associated with the early stages of multiple types of neurodegenerative disorders. Molecular factors that regulate neurite degeneration remain poorly understood, and existing methods for studying neurite degeneration have limited application and efficiency. Here, we identify modifications to a widely used procedure for analyzing neurite degeneration that reduce measurement error. Such methodological enhancements were incorporated into an ImageJ macro, thereby facilitating rapid and completely automated analysis of neurite degeneration using large sets of micrographs. Using a cell culture model of Parkinson’s disease (PD), we demonstrate that the macro facilitates more accurate and efficient neurite degeneration measurements while expanding the suitability of the analysis method for experiments involving dissociated cultures.

## Introduction

Neurite degeneration is a cellular process in which axons or dendrites undergo progressive dysfunction and morphologic changes, leading to a loss in neurite integrity. Neurite degeneration is an evolutionarily conserved process that is critical for the proper establishment of neural networks during the development of the central nervous system (Neukomm and Freeman, 2014). However, the process is also triggered in response to various forms of injury such as mechanical damage or exposure to drugs used for chemotherapy (Salvadores et al., 2017). Furthermore, neurite degeneration is associated with the progression of a variety of age-related neurodegenerative disorders, including Alzheimer’s disease, Parkinson’s disease (PD), Huntington’s disease, and amyotrophic lateral sclerosis. Interestingly, for all such neurodegenerative disorders, accumulating evidence suggests that neurite degeneration is a key, early-stage event that precedes neuronal death or onset of clinical symptoms (Serrano-Pozo et al., 2011; Adalbert and Coleman, 2013; Marangoni et al., 2014; Suzuki et al., 2020). Thus, studies exploring factors that regulate this cellular process may reveal novel targets for treating a variety of neurodegenerative conditions.

Investigating neurite degeneration is particularly important for understanding PD, a disease characterized by the accumulation of protein aggregates containing α-synuclein, progressive loss of dopaminergic neurons in the substantia nigra pars compacta (SNpc), and the development of a variety of motor and cognitive symptoms (Aarsland et al., 2017). Evidence from postmortem and human imaging studies indicates that α-synuclein aggregates are more abundant in neuronal fibers than in the cell bodies, and degradation of dopaminergic axons occurs to a greater extent than loss of dopaminergic neurons in the SNpc during the period leading to clinical onset (Tagliaferro and Burke, 2016). While the molecular interactions that contribute to the progressive deterioration of dopaminergic neurites remain incompletely understood, recent evidence indicates that neurite degeneration can be mediated by events independent of signaling cascades underlying neuronal death (Fukui, 2016; Geden and Deshmukh, 2016). Thus, studies which directly evaluate physiological underpinnings of neurite degeneration are critical to the development of therapies that impede early PD progression.

In response to the evidence of a central role for neurite degeneration in the progression of neurodegenerative disorders, a variety of methods have been developed to measure neurite degeneration using micrographs of cultured neurons. For example, multiple different methods have been established to measure neurite degeneration using software to detect decreased neurite number or length (Ofengeim et al., 2012; Johnstone et al., 2018). However, since such methods do not directly evaluate neurite fragmentation, interpretation of results to determine the impact of a variable on neurite degeneration can be confounded by potential effects of the variable on neurite outgrowth. Certain research groups have also measured neurite degeneration by subjectively scoring images of neurites based on morphologic features and the degree of apparent fragmentation (Zhai et al., 2003; Nakamura et al., 2016; Krishtal et al., 2017). However, this alternative method is time consuming and may introduce investigator error.

One method that facilitates objective and direct measurement of neurite fragmentation involves use of the Analyze Particles algorithm of ImageJ to measure the area of neurite fragments in micrographs of cultured cells. The measurements are used to calculate a Degeneration Index (DI), a scale between 0.0 and 1.0 reflecting the degree of neurite degeneration (Sasaki et al., 2009). While DI analyses can be used to detect varying degrees of neurite fragmentation, the accuracy of measurements is commonly limited by several sources of error, and application of the method has been primarily limited to explant cultures or cells grown in compartmentalized microfluidic devices. Moreover, DI analyses are time consuming when extrapolated to large image sets. Thus, we sought to overcome these limitations by modifying the DI analysis procedure to facilitate greater accuracy and efficiency, while expanding applicability.

In the present study, we identify several sources of error commonly associated with DI analyses and report methodological revisions that reduce the various forms of error. We incorporate the procedural enhancements in an ImageJ macro termed Automated Neurite Degeneration Index (ANDI) to completely automate DI analyses from large image sets. Operation of the ANDI Macro only requires software and files that are open source and freely available. Measurements obtained using ANDI benefit from increased accuracy while substantially reducing the time required to perform the analyses. Moreover, we demonstrate how the new analysis method can be applied to perform accurate and rapid measurement of neurite degeneration in a cell culture model of PD involving dissociated cells grown without compartmentalization. Collectively, these methodological enhancements may lead to novel insight into factors underlying an important cellular process associated with neurodegeneration.

## Materials and Methods

### Cell culture

Cultures of Lund human mesencephalic (LUHMES) cells were established in Lab Tek II eight-well chamber slides coated with poly-L-ornithine (PLO) and fibronectin, poly-D-lysine (PDL) and laminin, all four substrates, or no substrates, as indicated. To coat with PLO and fibronectin, slides were incubated overnight at room temperature with a solution containing 200 μg/ml PLO (Sigma-Aldrich, catalog #P3655). Following washes with sterile water (Avantor), the slides were incubated with 2 μg/ml fibronectin (Sigma-Aldrich, catalog #F0895) for 3 h at 37°C. Slides were then washed with sterile PBS (Corning Life Sciences, catalog #21-040-CV) and allowed to air-dry. To coat with PDL and laminin, slides were incubated at room temperature with a solution containing 100 ng/ml PDL (Sigma-Aldrich, catalog #P7280) and 10 μg/ml laminin (Corning, catalog #354232). Following an overnight incubation, the slides were washed with sterile PBS and allowed to air dry. To coat with all four substrates, the aforementioned procedures were performed sequentially. LUHMES cells (ATCC, RRID:CVCL_B056) were plated in the coated chamber slides at the indicated densities in differentiation medium consisting of DMEM/F12 (Invitrogen, catalog #11330057) supplemented with 2 mm glutamine (VWR), 1% (v/v) N-2 supplement, 1 μg/ml tetracycline (Sigma-Aldrich, catalog #87128-25G), 1 mm N6,2′-O-dibutyryladenosine cAMP (db-cAMP; Enzo Life Sciences, catalog #BML-CN125-0100), and 2 ng/ml glial cell line-derived neurotrophic factor (GDNF; R&D Systems, catalog #212-GD-010). Except where otherwise indicated, all cultures were plated at a density of 115,000 cells per well. A 50% volume media replacement was performed every 48 h until treatment. To prevent genetic drift, cell stocks that had been passaged six or fewer times were used for all experiments.

Experiments involving primary cultures of sympathetic neurons were approved by the Animal Care and Use Committee at Eastern Kentucky University or the University of Virginia. Superior cervical ganglia were dissected from postnatal day 0–6 C56Bl/6 mice, incubated at 37°C in an enzymatic dissociation buffer, washed with growth media to remove proteolytic enzymes, and triturated. Cell suspensions were plated at a density of 7000 cells per well in Lab Tek II eight-well chamber slides. Media were changed at day in vitro (DIV) 1, followed by media replacement every other day. Media were supplemented with cytosine arabinofuranoside (Ara-C, Millipore-Sigma) on DIV1–DIV3 to prevent the growth of non-neuronal cells, followed by removal of Ara-C from the media for at least 24 h before treatment as indicated. For experiments conducted at Eastern Kentucky University involving hydrogen peroxide treatment, the chamber slides were coated with 200 μg/ml PLO (Sigma-Aldrich, catalog #P3655), 2 μg/ml fibronectin, 100 ng/ml PDL (Sigma-Aldrich, catalog #P7280) and 10 μg/ml laminin (Corning, catalog #354232) before use, and dissociation was performed via 30-min incubation in buffer consisting of DMEM (ThermoFisher, catalog #11960-044) containing 0.025% trypsin (Corning Life Sciences, catalog #25-053-CI) and 0.3% collagenase (Worthington Biochemical, catalog #LS004176). Growth media for such experiments consisted of DMEM supplemented with 10% fetal bovine serum (Invitrogen, catalog #26140-079), 2 mm L-glutamine (Corning, catalog #25-005-CI), and 40 ng/ml nerve growth factor (Fisher Scientific, catalog #13-257-019); 5 μm Ara-C (Sigma-Aldrich) was used for removal of non-neuronal cells. For experiments conducted at the University of Virginia involving NGF withdrawal, cells were plated on chamber slides coated with 100 ng/ml PDL (Millipore Sigma) and 10 μg/ml laminin (Invitrogen), and dissociation was performed via 20-min incubation in buffer consisting of DMEM/F12 containing BSA (10 mg/ml, Sigma-Aldrich), collagenase type 2 (4 mg/ml, Worthington Biochemical), deoxyribonuclease 1 (Millipore Sigma), and hyaluronidase (Sigma-Aldrich). Growth media for such experiments consisted of DMEM supplemented with 10% fetal bovine serum (Invitrogen), 1 U/ml penicillin/streptomycin (Invitrogen), and 45 ng/ml of NGF (purified from salivary glands); 10 μm Ara-C was used for removal of non-neuronal cells.

### Cell treatments

Differentiated LUHMES cells were exposed to the indicated concentrations of 6-hydroxydopamine (6-OHDA; Sigma-Aldrich, catalog #162957) or vehicle solution on their fifth day in differentiation medium. Stocks of 6-OHDA were prepared in chilled PBS containing 0.02% ascorbate (Sigma-Aldrich, catalog #A5960), and aliquots were stored in light-protected vials under inert gas at −80°C. After 24 h of treatment, the cells were fixed by incubation in PBS containing 4% paraformaldehyde (PFA) for 25 min at room temperature. To determine the role of c-Jun N-terminal kinase (JNK) in neurite degeneration induced by oxidative stress, cultures were pretreated for 1 h with 10 μm SP600125 (Sigma-Aldrich, catalog #S5567) or vehicle solution before complete replacement of media with media containing 7.5 μm 6-OHDA, 7.5 μm 6-OHDA and 10 μm SP600125, or vehicle solutions. Stocks of SP600125 were prepared by dissolving the inhibitor in sterile dimethylsulfoxide (DMSO), and frozen aliquots were stored in light-protected vials filled with inert gas at −20°C. Treatments were performed such that cells were exposed to no greater than 0.1% DMSO.

Primary cultures of sympathetic neurons were treated on DIV5. For experiments involving neurite degeneration induced by hydrogen peroxide, the cultures were exposed to 500 μm hydrogen peroxide (Sigma-Aldrich, catalog #H1009-100ML) or vehicle solution consisting of sterile water (Cytiva, catalog #SH30529.02) for 24 h. For experiments involving NGF withdrawal, SCG cultures either received a media replacement with growth media or were subjected to NGF deprivation. SCG cultures to be deprived of NGF were rinsed three times with NGF-free growth media and then incubated in NGF-free growth media for 80 h without media changes. After treatment, the cells were fixed by incubation in 4% PFA in PBS for 30 min, followed by removal of the PFA by rinsing with PBS.

### Staining and fluorescence microscopy

For experiments with LUHMES cells, as well as the experiments involving sympathetic neurons treated with hydrogen peroxide, eight-well chamber slides containing fixed cells were permeabilized with 0.1% Triton X-100 in PBS and blocked for 1 h with 10% normal goat serum in PBS containing 0.1% Triton X-100. The slides were then incubated with primary antibody specific for βIII-tubulin (Covance; 1:1000, RRID:AB_2313773) in blocking solution at 4°C overnight. Subsequently, the slides were washed with PBS, incubated with a secondary antibody coupled to Alexa Fluor 488 (Thermo Fisher Scientific; 1:500, RRID: AB_142495) for 90 min at room temperature, and subjected to additional washes. For experiments involving tyrosine hydroxylase (TH) staining, the slides were subjected to an additional incubation in PBS containing 10% normal goat serum, 0.1% Triton X-100, and antibody specific for TH (Sigma-Aldrich, 1:400, AB152) at 4°C overnight. The slides were then washed with PBS, incubated with secondary antibody coupled to Alexa Fluor 568 (Thermo Fisher Scientific; 1:500, RRID: AB_1500889), and subjected to additional PBS washes. Following completion of immunolabeling for βIII-tubulin or TH, slides were incubated with 5 μg/ml 4′,6′-diamidino-2-phenylindole (DAPI; catalog #D9542) in PBS for 5 min. Slides were then washed with PBS, and coverslips were mounted using Fluoromount G mounting medium (Southern Biotech, catalog #0100-01).

For experiments involving sympathetic neurons subjected to NGF withdrawal, the slides were blocked for 1 h by incubation at room temperature in buffer consisting of 5% normal goat serum (Life Technologies) and 0.0005% Triton X-100 in PBS, followed by incubation in primary antibody solution containing mouse anti-mouse Tuj1 (1:1000, Covance) overnight at 4°C. Subsequently, slides were rinsed with PBS, incubated for 1 h in goat anti-mouse IgG Alexa Fluor 488 (1:800, Invitrogen) and 0.1 μg/ml Hoechst 33342 (Invitrogen) in blocking buffer, subjected to additional PBS rinses, and allowed to dry. The slides were mounted with Fluoromount-G (Southern Biotech, catalog #0100-01) and stored at room temperature.

Slides containing LUHMES cells were visualized with a 20× objective of a Nikon Eclipse II Ti-U Inverted Microscope System with a Lumencor Mira 4-Channel LED light source. 1280 × 1024-pixel images were captured using a Nikon DS-Qi1Mc 5 MP CCD Camera and NIS Elements 3.22.15 (Build 738). Except for where otherwise indicated, all phase-contrast images were captured using light intensity and exposure time settings that produced an average background intensity of 165 in 8-bit images. To reduce background shading or other inconsistencies, before each microscopy session an image of an empty well was captured and used for background correction during LUHMES cell imaging. Slides containing sympathetic neurons cultured at Eastern Kentucky University or at the University of Virginia were imaged using a Zeiss LSM 800 microscope system or a Zeiss LSM 980 microscope system, respectively. Images were captured at 20× magnification and 1024 × 1024 resolution using Zen software (Carl Zeiss Microscopy).

### Cell viability analysis

Differentiated LUHMES cell cultures were established on Lab Tek II eight-well chamber slides that were coated as indicated with various substrates. A 50% media change was performed every 48 h, and the cells were fixed with 4% PFA in PBS on the sixth day of differentiation. Slides containing fixed cells were subjected to staining with DAPI, and images of nuclear staining were captured with a Nikon Eclipse II Ti-U Inverted Microscope System and Nikon DS-Fi1 5 MP CCD Camera. Cells were scored as healthy or unhealthy by a blinded investigator based on the appearance of the nuclei. Cells with decreased nuclear area or nuclear fragmentation were scored as unhealthy. The number of healthy cells per image was scored for at least five fields of view per condition in each experiment.

### DI measurements

Traditional DI measurements were performed using a Fiji Is Just ImageJ (FIJI) package containing ImageJ 1.52p via a method involving widely cited parameters for DI analysis (Shin et al., 2012; Di Stefano et al., 2015; Sasaki et al., 2016; Loreto et al., 2020; Shin and Cho, 2020). Eight-bit images of differentiated LUHMES cells with well-separated axon tracts were subjected to binarization using the default binarization algorithm, with binary options configured to produce an image with black cells in the foreground and white background. The freehand drawing tool was used to manually trace all image regions featuring cell bodies, and all black pixels were converted to white in the traced regions. The Measure tool of ImageJ was applied to measure the total black area of the binarized images exclusively featuring neurites. The Analyze Particle tool was then applied using a size setting of 20–10,000 pixels and a circularity setting of 0.2–1.0 to detect neurite fragments. The summed area of all neurite fragments was then divided by the total black area to calculate the DI of the image.

For experiments related to optimization of size parameters for identification of neurite fragments, circularity criteria of 0.2–1.00 were used for all particle measurements. The Particle Remover plugin (version 2004/02/09), developed by Wayne Rasband, was obtained from https://imagej.nih.gov/. Where indicated, application of Particle Remover plugin using size parameters of 0–9 pixels and circularity parameters of 0.0–1.0 was performed to remove small, non-neurite objects from binarized images obtained from phase-contrast micrographs. Similarly, size parameters of 0–4 pixels and circularity parameters of 0–1.0 were used to remove small objects from binarized images obtained from fluorescence micrographs representing βIII-tubulin immunostaining. Unless otherwise indicated, before DI analysis all phase-contrast images were subjected to contrast enhancement by using ImageJ to apply a Lookup Table (LUT) with a minimum of 90 and maximum of 205.

### Measurement of artificial fragmentation

Cultures containing healthy LUHMES cells were fixed with 4% PFA on their sixth day of differentiation and immunolabeled for βIII-tubulin. Using a 20× objective, 1280 × 1024-pixel phase-contrast micrographs and fluorescence micrographs, each depicting identical fields of view, were captured from randomly sampled regions. A total of ten sets of images were captured from nine separate cultures. ImageJ was used to generate binarized copies of the original phase-contrast and fluorescence micrographs, as well as copies of phase-contrast micrographs that were binarized after contrast enhancement via application of an LUT with a minimum of 90 and maximum of 205. For each set of images, a blinded investigator identified a small, 150 × 150-pixel region of the original phase-contrast micrograph featuring exclusively healthy and intact neurites. The region size and coordinates were then stored in the region of interest (ROI) manager of ImageJ and used to perform DI measurements in analogous regions of binarized images from the same image set. The DI measurements were performed using the traditional method featuring fragment size parameters of 20 -10,000 pixels. Since all regions measured contained exclusively intact neurons, any fragments detected were considered artificial fragments.

### ANDI Macro

The ANDI Macro was written using ImageJ Macro language. The macro features a series of operations that enable the user to select paths for three directories, one directory containing images of neurons immunolabeled for a neuronal marker such as βIII-tubulin, a second directory containing corresponding images depicting nuclear staining, and a third directory designated for saving result files. The fragment areas and total neurite areas of the images in the selected directories are then calculated via a series of ImageJ operations. In brief, an image depicting nuclear labeling is opened, subjected to contrast enhancement, and binarized. The particle remover plugin is used to remove small, non-nuclear artifacts from the binarized image, and the binarized image is subjected to a series of dilations and erosions. A corresponding image of cells immunolabeled for βIII-tubulin (or other neuronal marker) is opened and subjected to contrast enhancement. The binarized image depicting enlarged cell nuclei is then subtracted from the image featuring cells immunolabeled for βIII-tubulin, thus generating an image featuring exclusively neurites. The neurite image is then binarized and subjected to removal of particles between 0 and 4 pixels. Neurite fragment area is calculated by applying the Analyze Particle algorithm to measure particles with a circularity between 0.2 and 1.0 and size between 5 and 10,0000 pixels. Total neurite area is calculated by applying the Measure tool to calculate the total black area. An image depicting the binarized neurite area with highlighted fragments is saved to a directory designated by the user, along with .csv files reporting the fragmented neurite area and total neurite area measurements.

In addition to the aforementioned operations, several additional operations are included in the macro to support ease-of-use and limit user error. These include functions that remove scale information from images and ensure that measurements are obtained in pixel units; configure the ImageJ color settings and binary options for appropriate image subtraction and binarization; verify that the selected directories for images of βIII-tubulin staining and DAPI staining contain an equal number of images; close previously used images to enhance processing time; clear results from previous analyses before performance of new analyses; and prevent errors associated with .ini file creation by Windows 10.

For experiments involving analyses of sympathetic neurons, the ANDI Macro was revised to include an operation that presents a dialogue box enabling the user to control the number of times that binarized images of nuclei will be dilated. The version of ANDI featuring this revision was termed ANDI v1.1. Changing the value in the dialogue box to a higher number increases the soma removal size, while changing the value to a lower number decreases the soma removal size. The default value is 12, which is the number of dilations used for all experiments involving LUHMES cells. A value of 16 was used to remove larger cell body regions during analyses of sympathetic neurons.

The ANDI Macro is open source and freely available for use or modification via the following URL: https://github.com/kraemerb/kraemerlab. Accurate operation of ANDI has been verified using multiple personal computers featuring the Windows 10 operating system. An image set featuring LUHMES cell cultures exhibiting various degrees of neurite degeneration is also available at the aforementioned web address and can be used to investigate accurate execution of the macro by other types of computers. The web address also provides access to a protocol describing how to use the macro.

### Time estimates

To evaluate the time required to manually perform DI measurements, phase-contrast micrographs depicting LUHMES cells that were treated with vehicle solution, 2.5 μm 6-OHDA, 5.0 μm 6-OHDA, or 7.5 μm 6-OHDA were subjected to DI scoring using previously-established methods (Kraemer et al., 2014). Each image was subjected to contrast enhancement and binarization. The freehand tool of ImageJ was then used to digitally remove all depicted cell bodies from the binarized images. The Analyze Particle tool was used to measure fragments with a circularity between 0.2 and 1.0 and a size between 20 and 10,000 pixels, and the Measure tool was used to determine the total neurite area. Values pertaining to fragmented neurite area and total neurite area were transferred to a spreadsheet in Microsoft Excel, and a mathematical operation in Excel was used to calculate the DI scores. After an image was opened with ImageJ, the time required to manually perform each of these procedures was measured. Average analysis times were calculated from 36 total images. The images were obtained from nine independent cell culture preparations, and analyses were performed using one image per treatment condition from each cell culture preparation. To determine the speed of DI measurements performed using the ANDI Macro, the same image sets were used to determine the time required to open the macro in ImageJ, run the macro operations, transfer the data to an Excel spreadsheet, and calculate the DI.

### Statistics

**Figure 1.**
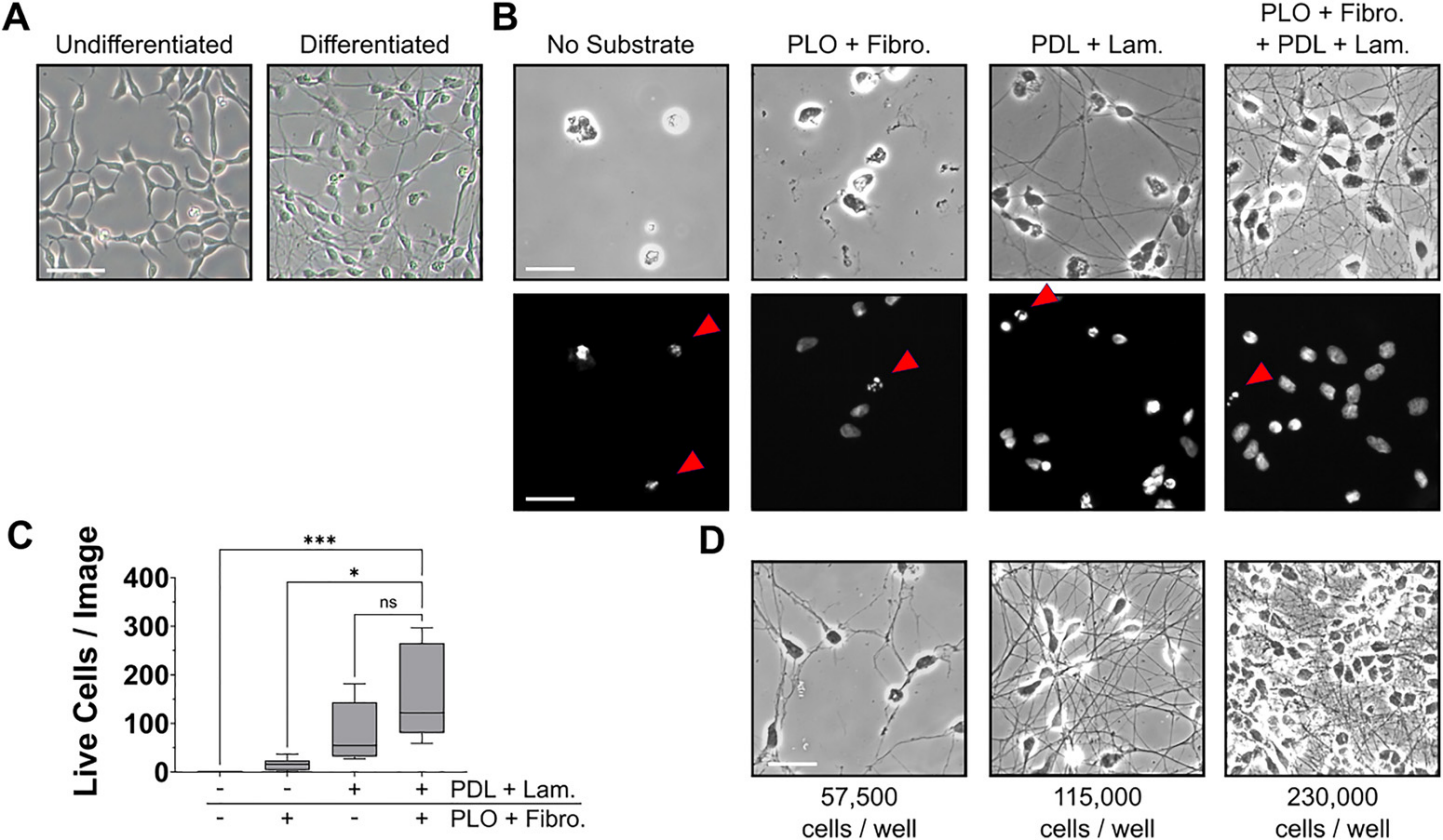
Optimization of LUHMES cell culture conditions for measurement of neurite degeneration. ***A***, Representative phase-contrast micrographs of LUHMES cells that were maintained in growth medium (left) or cultured in differentiation medium for 5 d (right). ***B***, Phase-contrast micrographs (top row) or images of DAPI staining (bottom row) of LUHMES cells that were cultured in differentiation medium for 5 d in Lab-Tek II chamber slides that were coated as indicated with no substrate, 200 μg/ml PLO and 2 μg/ml fibronectin, 100 ng/ml PDL and 10 μg/ml laminin, or all four substrates. Red arrowheads indicate nuclei that were considered as apoptotic because of low nuclear area or apparent nuclear fragmentation. ***C***, Quantification of live cells in cultures established as described in ***B***. The cells were fixed on their sixth day of differentiation with 4% PFA, and nuclei were labeled with DAPI. Five fluorescence micrographs depicting DAPI staining were captured per condition in each experiment, and cells were scored as healthy or dying based on the appearance of nuclei. Coating with all four substrates resulted in a significant increase in the mean number of live cells compared with cultures established in slides coated with PLO and fibronectin (*n *=* *6; *p* = 0.0437, Friedman test with Dunn’s correction for multiple comparisons). ***D***, Representative phase-contrast micrographs of LUHMES cell cultures established using different plating densities as indicated. The cells were plated in differentiation medium, and images were captured on the fifth day in culture. PLO, poly-L-ornithine; Lam.; laminin; PDL, poly-D-lysine; Fibro., fibronectin; *p < 0.05, ***p < 0.001; ns, not significant; *n*, number of experiments, each featuring an independent cell culture preparation. Scale bar:* *25 μm.

Quantitative comparisons are presented as box and whisker plots, where the middle line represents the median, boxes extend to the 25th and 75th percentiles, and whiskers depict the 5th and 95th percentiles. Means were statistically compared in GraphPad Prism 9.1.2 by repeated measures ANOVA with *post hoc* comparisons using a Šidák correction. Normality for quantitative data were tested using the Shapiro–Wilk test in IBM SPSS 26. When the assumption of normality was violated (Figs. 1*C*, 3*D*), group comparisons were conducted via nonparametric Friedman test with Dunn’s test performed for *post hoc* comparisons. Correlational analyses are depicted as scatterplots and include lines of best fit. Such relationships were analyzed using Pearson product moment correlations or Spearman’s ρ when the assumption of normality was violated. Normality of residuals for correlational analysis was assessed through visual inspection of corresponding Q-Q plots. Pearson product moment correlation coefficients were compared with one another using Steiger’s asymptotic z-test (Steiger, 1980; Lee and Preacher, 2013). No outliers were excluded. Statistical significance was accepted at *p* < 0.05. *P*-values are rounded to four decimal places and reported throughout the main text. Effect sizes and 95% confidence intervals are also reported for parametric analyses, with effect sizes described throughout the main text and confidence intervals listed in Table 1.

**Table 1:** Statistical table

Data	Data structure	Type of test	Power (95% confidence intervals)	
Fig. 1*C*	Non-normal	Friedman test with Dunn’s correction	Not applicable
Fig. 2*G*	Normal	ANOVA with Šidák correction	0 μm (vehicle) vs 2.5 μm 6-OHDA	−0.06461–0.01985
			0 μm (vehicle) vs 5.0 μm 6-OHDA	−0.3547 to −0.1754
			0 μm (vehicle) vs 7.5 μm 6-OHDA	−0.5079 to −0.3277
			2.5 μm 6-OHDA vs 5.0 μm 6-OHDA	−0.3429 to −0.1424
			2.5 μm 6-OHDA vs 7.5 μm 6-OHDA	−0.4865 to −0.3044
			5.0 μm 6-OHDA vs 7.5 μm 6-OHDA	−0.2632 to −0.04233
Fig. 3*D*	Non-normal	Friedman test with Dunn’s correction	Not applicable
Fig. 3*E*	Normal	ANOVA with Šidák correction	0 μm (vehicle), Phase vs Fluorescence	0.1055–0.1741
			2.5 μm 6-OHDA, phase vs fluorescence	0.1213–0.1985
			5.0 μm 6-OHDA, phase vs fluorescence	0.1462–0.2764
			7.5 μm 6-OHDA, phase vs fluorescence	−0.1731–0.003029
Fig. 4*C*	Normal	ANOVA with Šidák correction	0 μm (vehicle), with particle removal vs without particle removal	0.005456–0.02108
			2.5 μm 6-OHDA, with particle removal vs without particle removal	0.004059–0.03360
			5.0 μm 6-OHDA, with particle removal vs without particle removal	0.03510–0.1167
			7.5 μm 6-OHDA, with particle removal vs without particle removal	0.09786–0.1797
Fig. 4*D*	Normal	ANOVA with Šidák correction	0 μm (vehicle), with particle removal vs without particle removal	−0.002760–0.001501
			2.5 μm 6-OHDA, with particle removal vs without particle removal	−0.003088–0.002654
			5.0 μm 6-OHDA, with particle removal vs without particle removal	−0.04534–0.01876
			7.5 μm 6-OHDA, with particle removal vs without particle removal	−0.1022–0.02869
Fig. 5*E*	Non-normal	Spearman’s rho	0.9604–0.9901
Fig. 5*F*	Normal	Pearson’s *r*	0.786–0.941
Fig. 5*G*	Normal	Pearson’s *r*	0.978–0.994
Fig. 6*C*	Normal	ANOVA with Šidák correction	Veh:ANDI (v1.1) vs Veh:Manual	−0.0005736–0.004670
			H_2_O_2_:ANDI (v1.1) vs H_2_O_2_:Manual	−0.08603–0.07370
			Veh:ANDI (v1.1) vs H_2_O_2_:ANDI (v1.1)	−0.7698 to −0.09204
			Veh:Manual vs H_2_O_2_:Manual	−0.7422 to −0.1360
Fig. 6*E*	Normal	Repeated measures ANOVA	0.04244–0.07132
Fig. 6*G*	Non-normal	Spearman’s rho	0.9186–0.9822
Fig. 7*B*	Normal	ANOVA with Šidák correction	0 μm (vehicle), pretreatment with vehicle vs SP600125	−0.006446–0.01094
			7.5 μm 6-OHDA, pretreatment with vehicle vs SP600125	0.4796–0.8076

Table indicating the data structure (normal or non-normal distribution), type of statistical test, and 95% confidence intervals associated with the experiments described in this article.

### Data availability

Additional data pertaining to the present article will be shared upon reasonable request.

### Code accessibility

The code/software described in the paper is freely available online at https://github.com/kraemerb/kraemerlab. The code is available as Extended Data 1.

10.1523/ENEURO.0327-21.2022.ed1 Extended Data 1 ANDI v1.1 script for image processing and DI analysis using ImageJ. Download Extended Data 1, ZIP file.

## Results

### Establishment of a cell culture model of neurite degeneration associated with PD

To develop a method for investigating neurite degeneration in a cell culture model of PD, we used cultured LUHMES cells, a population of conditionally immortalized, human mesencephalic cells. Upon differentiation, LUHMES cells become postmitotic, adopt a gene expression profile characteristic of mature dopaminergic neurons, and develop a neuronal morphology with neurites that extend up to 500 μm in length (Fig. 1*A*; Lotharius et al., 2005; Scholz et al., 2011; Kraemer et al., 2021). Because of the superior optical qualities of glass compared with plastic, the cultures were established on glass slides containing eight media chambers. Based on previous reports (Lotharius et al., 2005; Scholz et al., 2011; Kurowska et al., 2014), we initially attempted to establish cultures on chamber slides coated with PLO and fibronectin; however, such cultures exhibited adhesion deficits and low viability. As a result, we tested whether coating with additional substrates would improve the viability of LUHMES cell cultures established on glass surfaces. LUHMES cell cultures were established on slides that were either uncoated or coated with PLO and fibronectin, PDL and laminin, or a combination of all four substrates. Analysis of viable nuclei by fluorescence microscopy revealed that slide coating significantly affected the viability of the cultures [Friedman χ^2^(3) = 18, *p *<* *0.0001], with *post hoc* multiple comparison analyses revealing that slides coated with a combination of all four aforementioned substrates yield a significantly higher rate of viable cells when compared with slides only coated with PLO and fibronectin (*p *=* *0.0437; Fig. 1*B*,*C*).

After identifying the optimal coating substrates for culturing LUHMES cells on glass slides, we evaluated the effects of cell density on the suitability of the cultures for neurite degeneration measurements. LUHMES cells were cultured in differentiation medium with plating densities of 57,500 cells per well, 115,000 cells per well, or 230,000 cells per well, and on their fifth day of differentiation the cells were fixed with 4% PFA and imaged by phase-contrast microscopy. A density of 57,500 cells per well resulted in cultures with poorly networked neurites and irregular neurite morphology, while cultures established with a plating density of 230,000 cells, although viable, contained neurite densities too high for accurate neurite measurement. The intermediate cell density of 115,000 cells per well provided high viability and well-separated axon tracts conducive to accurate neurite degeneration measurements (Fig. 1*D*).

**Figure 2.**
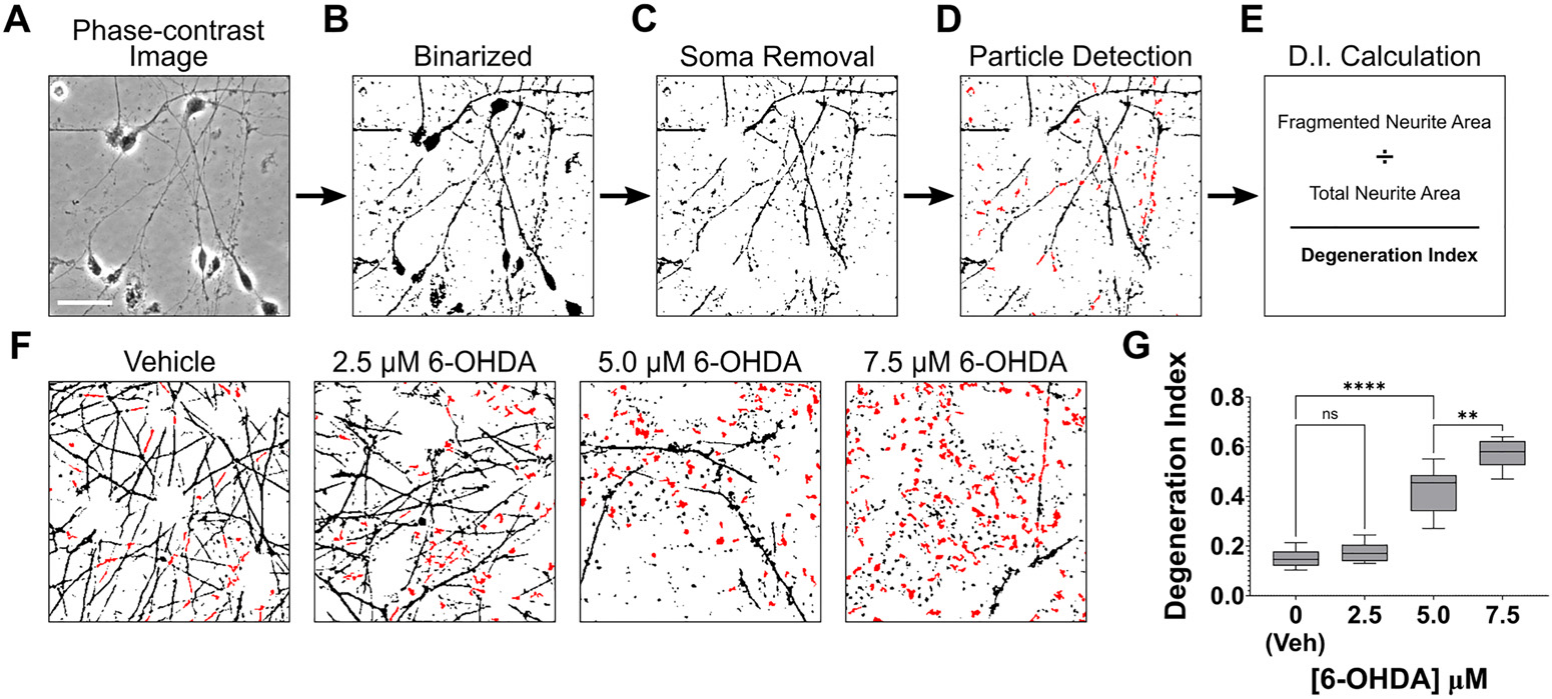
Measurement of neurite degeneration in LUHMES cell cultures exposed to 6-OHDA using the traditional DI analysis method. ***A–E***, Schematic representing the steps through which DI analyses are traditionally performed. Phase-contrast images (***A***) are binarized to yield an image featuring black cells and white background (***B***). Any cell bodies in the image are then removed to generate an image exclusively featuring neurites (***C***). The Analyze Particles tool of ImageJ is then used to detect neurite fragments with a size of 20–10,000 pixels and circularity of 0.2–1.0 pixels. The detected fragments are highlighted in red (***D***). The summed area of the neurite fragments is divided by the total neurite area, calculating by measuring the area of all black pixels, to yield a DI value (***E***). ***F***, Binarized images of LUHMES cells with cell bodies removed and neurite fragments highlighted in red. The LUHMES cells were differentiated for 5 d and then exposed to the indicated concentrations of 6-OHDA or vehicle solution for 24 h. The cells were then fixed with 4% PFA, and phase-contrast micrographs were subjected to DI analysis using the traditional procedure described in ***A–E***. ***G***, Quantification of neurite degeneration using phase-contrast micrographs of LUHMES cells that were exposed to various concentrations of 6-OHDA and analyzed as described in 2F. Exposure to 6-OHDA resulted in a significant and dose-dependent increase in neurite degeneration (*n *=* *9, ANOVA with Šidák correction for multiple comparisons). veh, vehicle; **p < 0.01, ****p < 0.0001; *ns*, not significant; *n*, number of experiments, each featuring an independent cell culture preparation. Scale bar: 25 μm.

To model PD-associated neurite degeneration *in vitro*, we exposed differentiated LUHMES cells to 6-OHDA, a widely used neurotoxin that promotes oxidative stress and degeneration of catecholinergic neurons (Bové and Perier, 2012). Exposure of differentiated LUHMES cells to 6-OHDA promoted a dose-dependent increase in neurite degeneration, with 5.0 μm 6-OHDA and 7.5 μm 6-OHDA inducing moderate and severe neurite degeneration, respectively (Fig. 2*F*). To quantify neurite degeneration in cultures of LUHMES cells exposed to 6-OHDA, we used a widely cited method in which phase-contrast images of cultures are binarized, cell bodies in the images are digitally removed, and the particle analyzer algorithm of ImageJ is applied to measure the area of neurite fragments. The fragmented neurite areas are then summed and divided by the total neurite area to determine a DI (Fig. 2*A–E*; Sasaki et al., 2009; Kraemer et al., 2014; Di Stefano et al., 2015; Hill et al., 2018; Geisler et al., 2019). As expected, exposure of differentiated LUHMES cells to 6-OHDA caused a dose-dependent increase in the DI (*F*_(3,24)_ = 119.8, *p *=* *0.0001, *R*^2^ = 0.9374; Fig. 2*G*). However, during the analyses of cells exposed to 6-OHDA we observed several issues that appeared to negatively impact the accuracy and efficiency of DI measurements. Thus, we sought to optimize this common method for performing DI measurements, which we henceforth refer to as the traditional DI method, to generate a protocol facilitating more effective measurement of neurite fragmentation.

### Reducing artificial fragmentation associated with micrograph binarization

**Figure 3.**
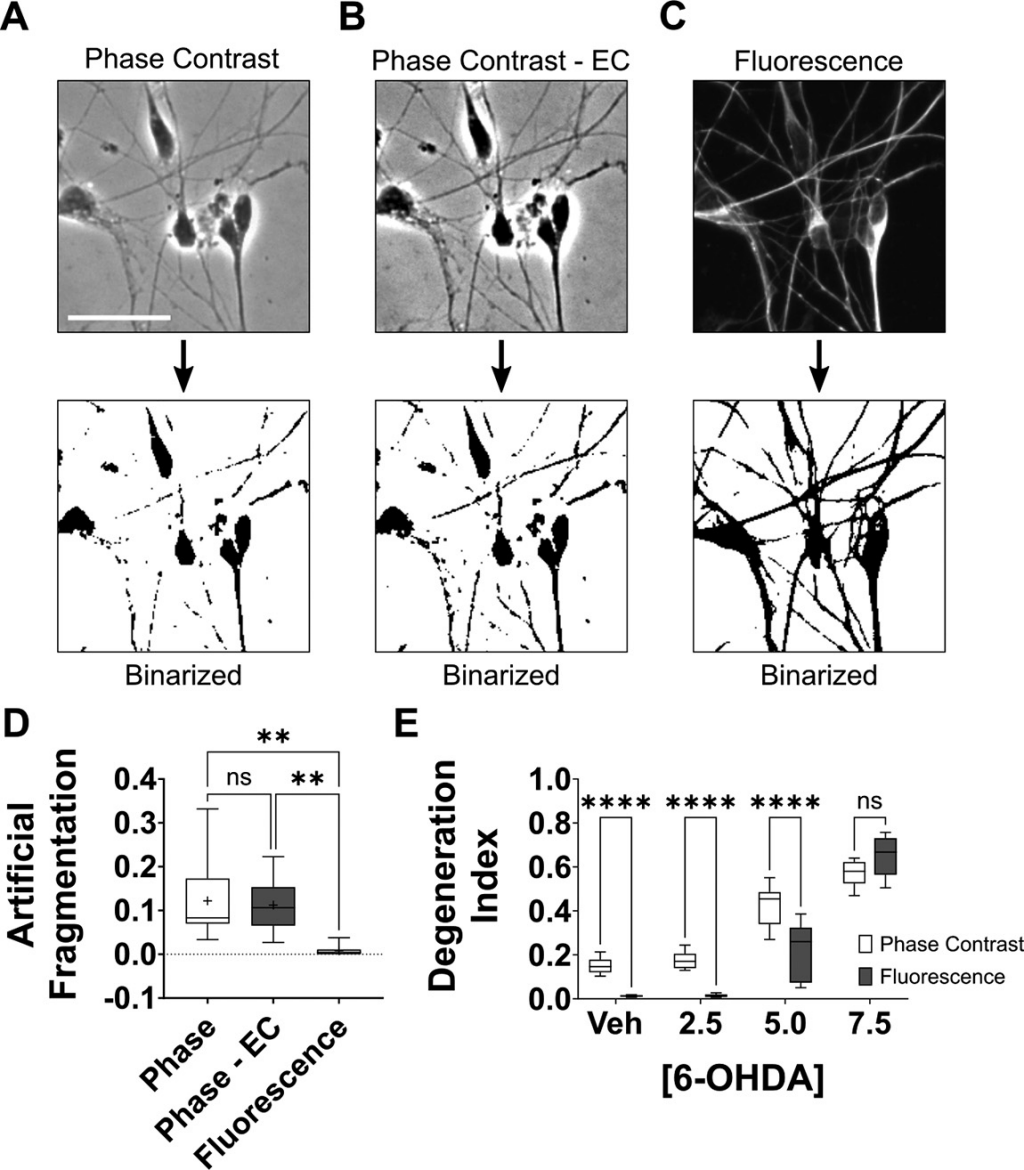
Reducing DI measurement error associated with binarization of phase-contrast micrographs. ***A***, Representative phase-contrast micrograph (top) depicting LUHMES cells with intact neurites that yields an image featuring cells with fragmented neurites upon binarization (bottom). ***B***, The phase-contrast image depicted in ***A*** was subjected to contrast enhancement using a LUT with a minimum of 90 and a maximum of 205 (top). The image was then subjected to binarization, yielding an image with comparatively greater neurite area yet still featuring fragmented neurites (bottom). ***C***, The cells displayed in ***A***, ***B*** were imaged by fluorescence microscopy following immunolabeling for βIII-tubulin (top). Binarization of the fluorescence micrograph yields an image featuring a higher proportion of intact neurites. ***D***, Quantification of artificial fragmentation in binarized images generated from images of healthy LUHMES cells with intact neurites. The binarized images were generated from original phase-contrast images (Phase), from phase-contrast images that were subjected to contrast enhancement (Phase – EC), or from fluorescence micrographs featuring an identical field of view and depicting immunofluorescence labeling for βIII-tubulin (Fluorescence). Fluorescence micrographs yielded binarized images with significantly reduced artificial fragmentation (*n *=* *10 images from 9 independent cell cultures; Friedman test with Dunn’s correction for multiple comparisons). ***E***, DI measurements obtained from the phase-contrast micrographs generated as described in Figure 2*F*,*G*, white bars, or images from identical fields of view depicting immunofluorescence labeling for βIII-tubulin (gray boxes). The analyses were performed using the traditional procedures described in Figure 2*A–E*. DI scores of cells featuring intact neurites (vehicle or 2.5 μm 6-OHDA treated) were significantly lower when obtained from fluorescence micrographs than when obtained from corresponding phase-contrast micrographs (*n *=* *9 experiments featuring independent cell culture preparations, ANOVA with Šidák correction for multiple comparisons). EC, enhanced contrast; veh, vehicle; **p < 0.01, ****p < 0.0001; *ns*, not significant. Scale bar:* *25 μm.

While using the traditional DI method, we frequently observed an issue in which intact neurites in phase-contrast images appeared fragmented in their corresponding binarized image (Fig. 3*A*). This artificial fragmentation primarily affected neurites with low prominence. Thus, we investigated whether the issue could be reduced by procedures that enhance image contrast. To improve image contrast, we first captured images of neurites using a variety of light intensities to determine the level of image brightness that would produce optimal contrast. Images captured with the optimal light intensity were then subjected to further contrast enhancement by using ImageJ to apply a LUT with minimum and maximum intensity values of 90 and 205, respectively. Incorporation of these contrast enhancements provided a moderately more accurate representation of the neurites in binarized images generated from phase-contrast images (Fig. 3*B*). Nevertheless, quantitative analysis of binarized images of healthy neurites indicated that the contrast enhancements did not significantly decrease artificial fragmentation (*p *=* *0.9999; Fig. 3*D*). Thus, we next investigated whether use of immunofluorescence imaging would produce images with superior contrast and thereby facilitate production of more accurate binarized neurite images with reduced artificial fragmentation. The slides described in Figure 2, featuring fixed LUHMES cells exposed to various concentrations of 6-OHDA, were immunostained for the cytoskeletal protein βIII-tubulin, imaged using fluorescence microscopy, and analyzed by a blinded investigator for artificial fragmentation (Fig. 3*C*). Quantitative analysis of binarized images obtained from fluorescence micrographs and corresponding phase-contrast micrographs revealed that the type of microscopy affected levels of artificial fragmentation [Friedman χ^2^(3) = 15.44, *p *<* *0.0001]. Specifically, fluorescence micrographs produced binarized images with significantly reduced artificial fragmentation compared with those from phase-contrast micrographs (*p = *0.0016; Fig. 3*D*). As a result, images of healthy cultures yielded significantly lower DI scores when fluorescence micrographs were used rather than phase-contrast micrographs (*F*_(1,8)_ = 131.4, *p = *0.0001, *R*^2^ = 0.1442; Fig. 3*E*). Moreover, a notable overall trend is that the fold change between mean DI values associated with healthy (vehicle-treated) cultures and cultures exhibiting neurite degeneration (5.0 or 7.5 μm 6-OHDA-treated) were greater when measured using fluorescence micrographs than when obtained from phase-contrast micrographs (Fig. 3*E*). This occurred despite fluorescence micrographs of cultures with moderate neurite degeneration (induced by exposure to 5 μm 6-OHDA) having significantly lower DI scores than corresponding phase images because of the reduction in artificial fragmentation (*p *=* *0.0001, *d *=* *0.6312).

### Optimizing parameters for fragmented neurite and total neurite detection

**Figure 4.**
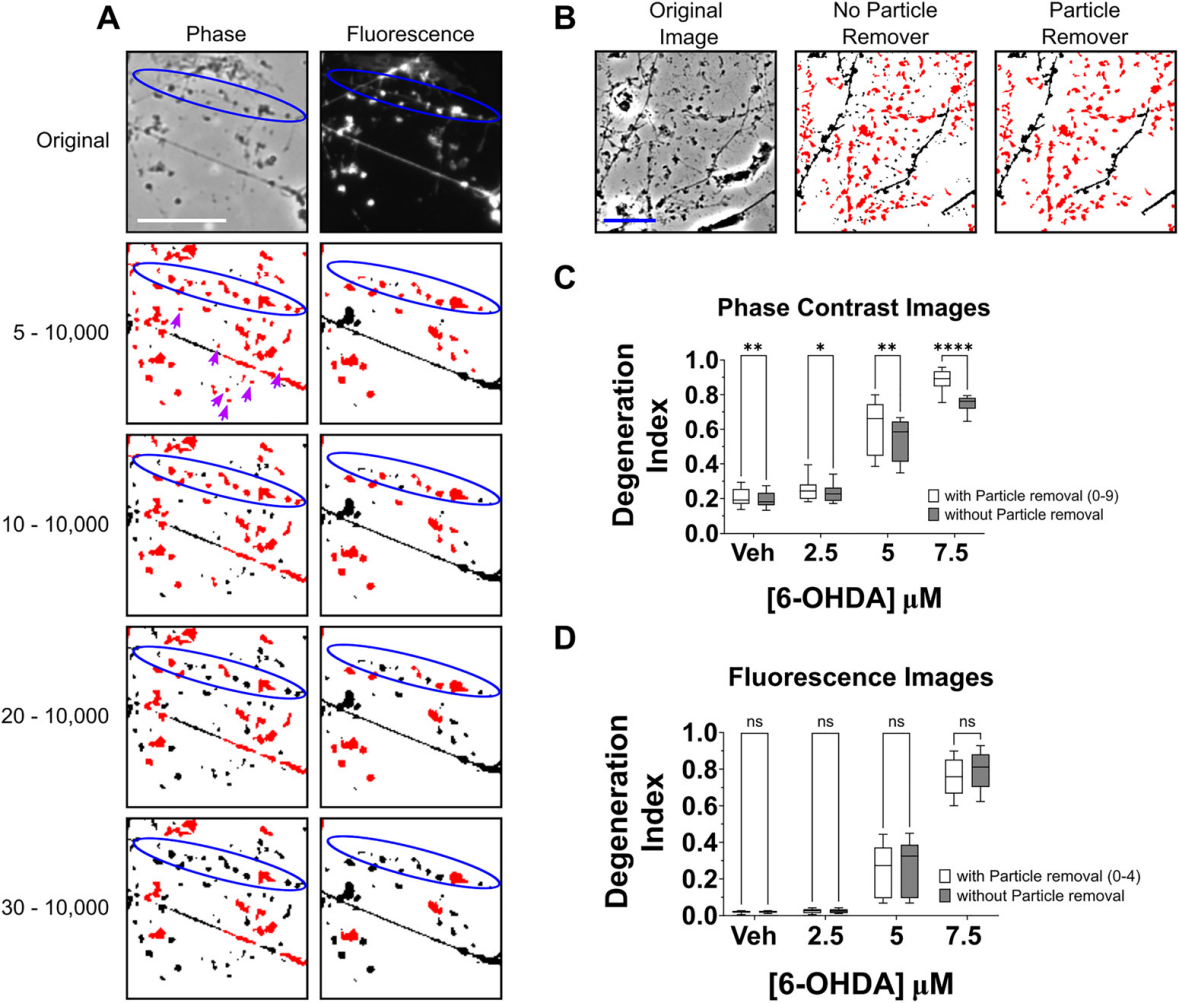
Refinement of parameters associated with fragmented neurite detection. ***A***, Phase-contrast (left) and fluorescence (right) micrographs depicting a fragmented neurite (circled in blue) were binarized and subjected to particle analysis using the indicated pixel size parameters for neurite fragment detection. Parameters of 5–10,000 observably yielded the most accurate and sensitive detection of fragments in binarized images obtained from fluorescence micrographs. However, similar particle analysis parameters resulted in the erroneous detection of non-neurite debris (purple arrows) as neurite fragment in binarized images obtained from phase-contrast micrographs. Thus, particle analysis size parameters of 10–10,000 pixels are recommended for DI measurements obtained from phase-contrast micrographs. ***B***, Representative images depicting LUHMES cells that were exposed to 5 μm 6-OHDA for 24 h. The phase-contrast micrograph (left) was binarized and subjected to DI analysis using fragment size parameters of 10–10,000 pixels. The neurite fragment detection was performed either without any additional image processing (middle image) or following removal of small particles <10 pixels in size (right image). ***C***, ***D***, Quantification of neurite degeneration using phase-contrast micrographs (***C***) of LUHMES cells that were generated as described in Figure 2*F*,*G* or corresponding fluorescence micrographs depicting immunofluorescence labeling for βIII-tubulin (***D***). The analyses of phase-contrast micrographs were performed using fragment size parameters of 10–10,000 pixels, while fluorescence micrographs were analyzed using fragment size parameters of 5–10,000 pixels. The analyses were performed using either binarized images without any additional image processing (gray bars) or following removal of all objects smaller than neurite fragments from the image (white bars). Removal of small particles significantly increased DI scores obtained from phase-contrast images but had no significant effect on DI scores obtained from fluorescence micrographs (*n *=* *9 experiments featuring independent cell culture preparations, ANOVA with Šidák correction for multiple comparisons). veh, vehicle; *p <* *0.05, **p < 0.01, ****p<0.0001; *ns*, not significant; *n*, number of experiments, each featuring an independent cell culture preparation. Scale bar:* *20 μm.

The traditional method for performing DI measurements involves use of the Analyze Particles plugin for ImageJ with size parameters of 20−10,000 pixels to detect neurite fragments. However, we observed that a large proportion of neurite fragments were undetected by the particle analyzer when using these widely cited parameters (Shin et al., 2012; Di Stefano et al., 2015; Sasaki et al., 2016; Loreto et al., 2020; Shin and Cho, 2020) to measure neurite degeneration in phase-contrast micrographs of LUHMES cells exposed to 6-OHDA, generated as described in Figure 2. Thus, we reanalyzed images from that dataset using different minimum size limits for particle detection to determine the optimal parameters for identification of neurite fragments. From qualitative assessment of binarized images generated from phase-contrast micrographs, size parameters of 5–10,000 pixels enhanced the detection of neurite fragments, but were also associated with misidentification of background noise, image artifacts, or other small non-neurite material as neurite fragments. Compared with the widely used parameters of 20–10,000 pixels, however, parameters of 10–10,000 pixels enhanced detection of neurite fragments without a noticeably large increase in false positives (Fig. 4*A*, left). Similar analyses were also performed with binarized images obtained from fluorescence micrographs of LUHMES cells immunolabeled for βIII-tubulin. Particle analyzer size parameters of 5–10,000 pixels facilitated the most sensitive detection of neurite fragments and, since the fluorescence micrographs featured less small debris, such parameters did not noticeably increase misidentification of non-neurite material as neurite fragment (Fig. 4*A*, right).

The traditional method for performing DI measurements, by using particle analyzer size parameters of 20–10,000 pixels, excludes objects smaller than 20 pixels from measurements of neurite fragments. However, such small objects are typically erroneously included in the total neurite area measurements, since the traditional DI measurement method involves calculating total neurite area by summing all black pixels in the binarized images. To overcome this issue, we used the Particle Remover plugin for ImageJ, written by ImageJ developer Wayne Rasband, to remove small, non-neurite objects from binarized images. Application of this plugin while performing DI measurements using phase-contrast micrographs of LUHMES cells exposed to 6-OHDA, generated as described in Figure 2, resulted in a significant increase in DI measurements across all concentrations of neurotoxin exposure (*F*_(1,8)_ = 83.43, *p = *0.0001, *R*^2^ = 0.0416). Furthermore, a significant interaction indicated that the increase in DI caused by the use of the particle remover became stronger at higher concentrations (*F*_(1.881,15.05)_ = 59.97, *p *=* *0.0001, *R*^2^ = 0.0094; Fig. 4*B*,*C*). We also imaged the same slides of cells by fluorescence microscopy and assessed the effects of small particle removal on DI measurements obtained from fluorescence micrographs. While small particle removal provides DI values that are theoretically more accurate, such values on average did not significantly differ from those obtained without use of the particle remover plugin when measured using fluorescence micrographs (*F*_(1,8)_ = 2.613, *p *=* *0.1446, *R*^2^ = 0.0013; Fig. 4*D*).

### Automation of DI measurements from fluorescence micrographs

**Figure 5.**
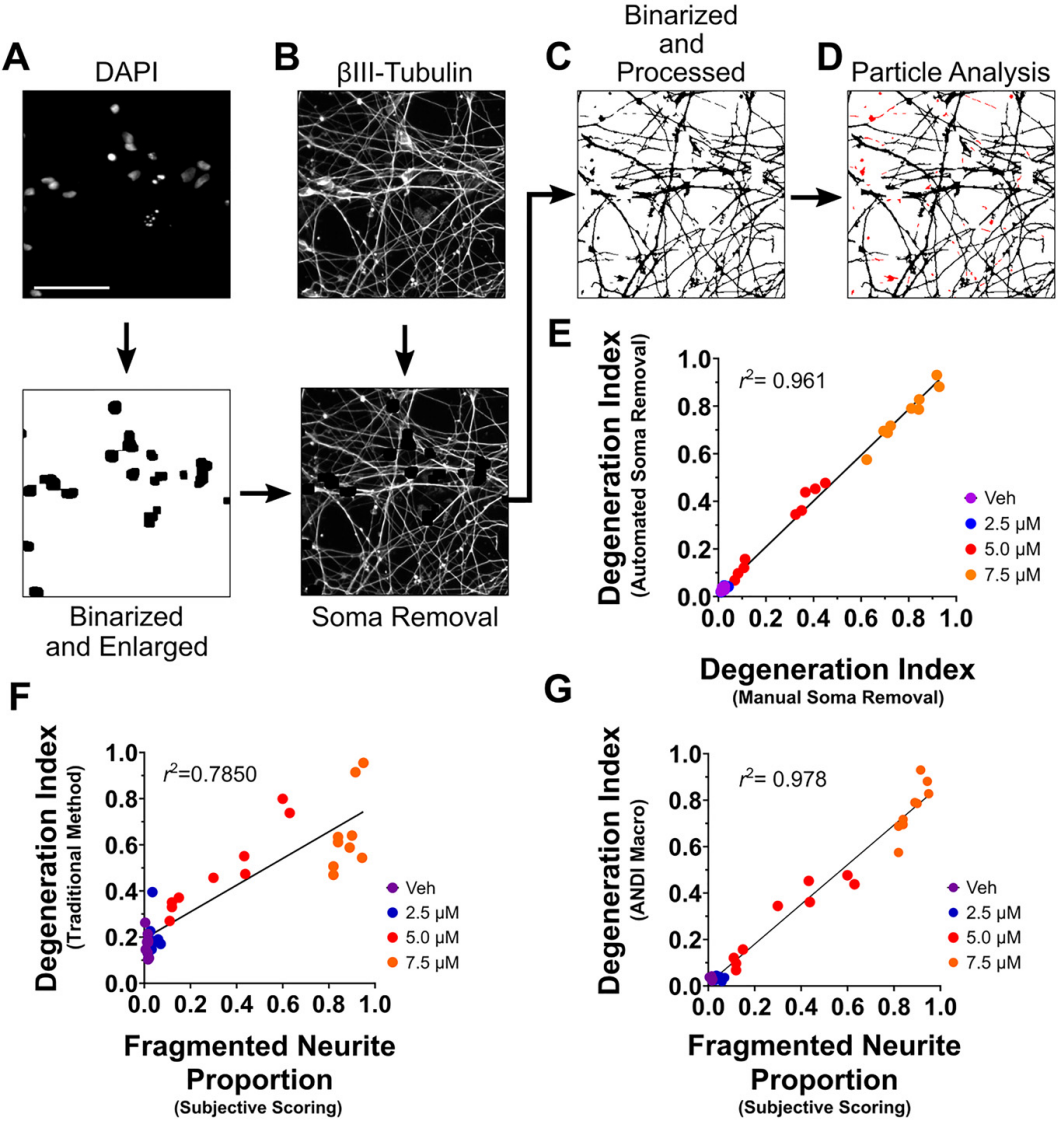
Accurate and automated measurement of neurite degeneration using the ANDI Macro. ***A–D***, Schematic depicting the major operations performed by the ANDI Macro. Images of DAPI-stained nuclei (***A***, top image) are binarized and subjected to multiple dilation and erosion operations, yielding an image with black regions that have been enlarged to encompass the size of a typical cell soma (***A***, bottom image). The image is then subtracted from a corresponding fluorescence micrograph depicting neurons immunolabeled for βIII-tubulin (***B***, top image), thereby producing an image exclusively featuring neurites (***B***, bottom image). The neurite image is binarized and subjected to additional processing with the Particle Remover plugin (***C***). The area of black pixels is summed to calculate the total neurite area, and the Analyze Particles tool is applied to measure neurite fragments with a size between 5 and 10,000 pixels (***D***). ***E***, Plot displaying the correlation between DI measurements obtained from images subjected to automated soma removal and DI scores from images subjected to manual soma removal. The fluorescence micrographs of LUHMES cells exposed to various concentrations of 6-OHDA, generated as described in Figure 3*E*, were subjected to the operations of the ANDI Macro related to soma removal using the freehand tool of ImageJ. The Particle Remover plugin was then applied to remove small objects less than five pixels in size from the images, the Measure tool was used to measure the total neurite area, and the Analyze Particles tool was applied to measure the area of fragments between 5 and 10,000 pixels. DI scores obtained from images subjected to automated soma removal correlated strongly with DI measurements from images subjected to manual soma removal (*n *=* *36, Spearman’s ρ, *r*_(34)_ = 0.980, *r*^2^ = 0.961). ***F***, Phase-contrast images of LUHMES cells that were exposed to various concentrations of 6-OHDA, generated as described in Figure 2*F*,*G*, were subjected to blinded scoring based on the proportion of neurite area that appears fragmented, with a value of 1.0 representing 100% neurite fragmentation and a value of 0 representing no neurite fragmentation. A correlation plot depicting the subjective scores and the DI measurements obtained using traditional DI analysis procedures (as described in Fig. 2) is displayed (*n *=* *36, Pearson’s *r* = 0.886, *r*^2^ = 0.785). ***G***, Plot depicting the correlation between the subjective scores of phase-contrast micrographs described in ***F*** and DI measurements obtained from corresponding fluorescence micrographs using the ANDI Macro. The correlation between subjective scores and DI values calculated using the ANDI Macro was significantly greater than the correlation between subjective scores and DI values obtained using traditional procedures (Steiger’s asymptotic z-test, z = 6.626, *p* < 0.001). veh, vehicle; *n*, number of experiments, each featuring an independent cell culture preparation. Scale bar:* *40 μm.

Performance of DI measurements using the traditional method requires tedious and time-consuming image processing, use of the freehand tool to digitally remove individual cell bodies, and measurement of fragmented neurite and total neurite areas from each image. Thus, we sought to enhance the efficiency of the method by writing a macro that fully automates DI analyses. To automate soma removal, the macro, titled ANDI, executes the binarization of images of cell nuclei, labeled via the nuclear stain DAPI, followed by modification of the binarized image such that thresholded nuclear regions are enlarged to encompass the entire region of the soma. Such images with binarized and enlarged nuclei are then subtracted from corresponding fluorescence micrographs of neurons immunolabeled for βIII-tubulin, generating an image exclusively featuring neurites (Fig. 5*A*,*B*). To adjust for variable nuclear sizes caused by nuclear shrinkage or fragmentation associated with neurodegeneration, the binarized images of nuclei are enlarged via a combination of dilations and erosions that combine nuclear fragments and expand binarized regions to occupy an area larger than the somas of differentiated LUHMES cells. Images exclusively containing neurites are then binarized and subjected to DI analysis via a series of automated functions (Fig. 5*C*,*D*). To evaluate the accuracy of the macro operations associated with soma removal from images, we compared the DI scores obtained following manual removal of cell bodies from fluorescence micrographs using the freehand tool of ImageJ (as previously depicted in Fig. 4*D*), to DI scores generated from analyses of identical images following automated cell body removal using the soma removal operations featured in the ANDI Macro. Our analyses revealed that DI scores calculated using automated cell body removal correlated nearly identically with DI measurements obtained from images subjected to manual soma removal (*r*_(34)_ = 0.980, *p *<* *0.0001; Fig. 5*E*).

To increase the accuracy of the analyses, we incorporated several of the previously described adjustments that enhance DI measurements into ANDI. The macro features contrast enhancement operations, use of the Particle Remover plugin to remove non-neurite matter from neurite images and non-nuclear matter from images of nuclei, and detection of neurite fragments using size parameters of 5–10,000 for the Analyze Particles plugin. We hypothesized that, collectively, the use of fluorescence microscopy, contrast enhancement operations, removal of small objects with the particle remover, and optimized parameters for particle detection would contribute to more accurate measurements of neurite degeneration. To test this hypothesis, the phase-contrast images of LUHMES cells exposed to various concentrations of 6-OHDA, generated as described in Figure 2, were analyzed by blind and subjective scoring using a value range between 0.0 and 1.0, with values representing the apparent proportion of fragmented neurite area. We then evaluated correlation between the subjective scores and DI measurements obtained via the traditional method, as well as correlation between the subjective scores and DI measurements obtained using the ANDI Macro. While DI measurements obtained using the traditional method correlated with subjective scores (*r*^2^ = 0.7850, *p* <0.0001; Fig. 5*F*), the ANDI Macro yielded measurements that correlated much more strongly with subjective ratings (*r*^2^ = 0.978, *p* < 0.0001; Fig. 5*G*). Direct statistical comparisons revealed that scores obtained via the ANDI Macro correlated significantly stronger with subjective ratings than do scores obtained via the traditional method (z = 6.626, *p *<* *0.0001; Fig. 5*F*,*G*). Further analyses revealed that the macro facilitates performance of DI measurements with robust and significant improvement in time efficiency. DI analyses performed using the ANDI Macro required an average time of 12.75 s per image analyzed, compared with an average time of 5.27 min per image analyzed via the traditional method [Friedman χ^2^(1) = 9, *p* = 0.0027]. Altogether, these results indicate that ANDI yields rapid generation of DI measurements that, compared with scores obtained via the traditional method, more closely approximate the degeneration that investigators perceive from qualitative analysis of neurite images.

**Figure 6.**
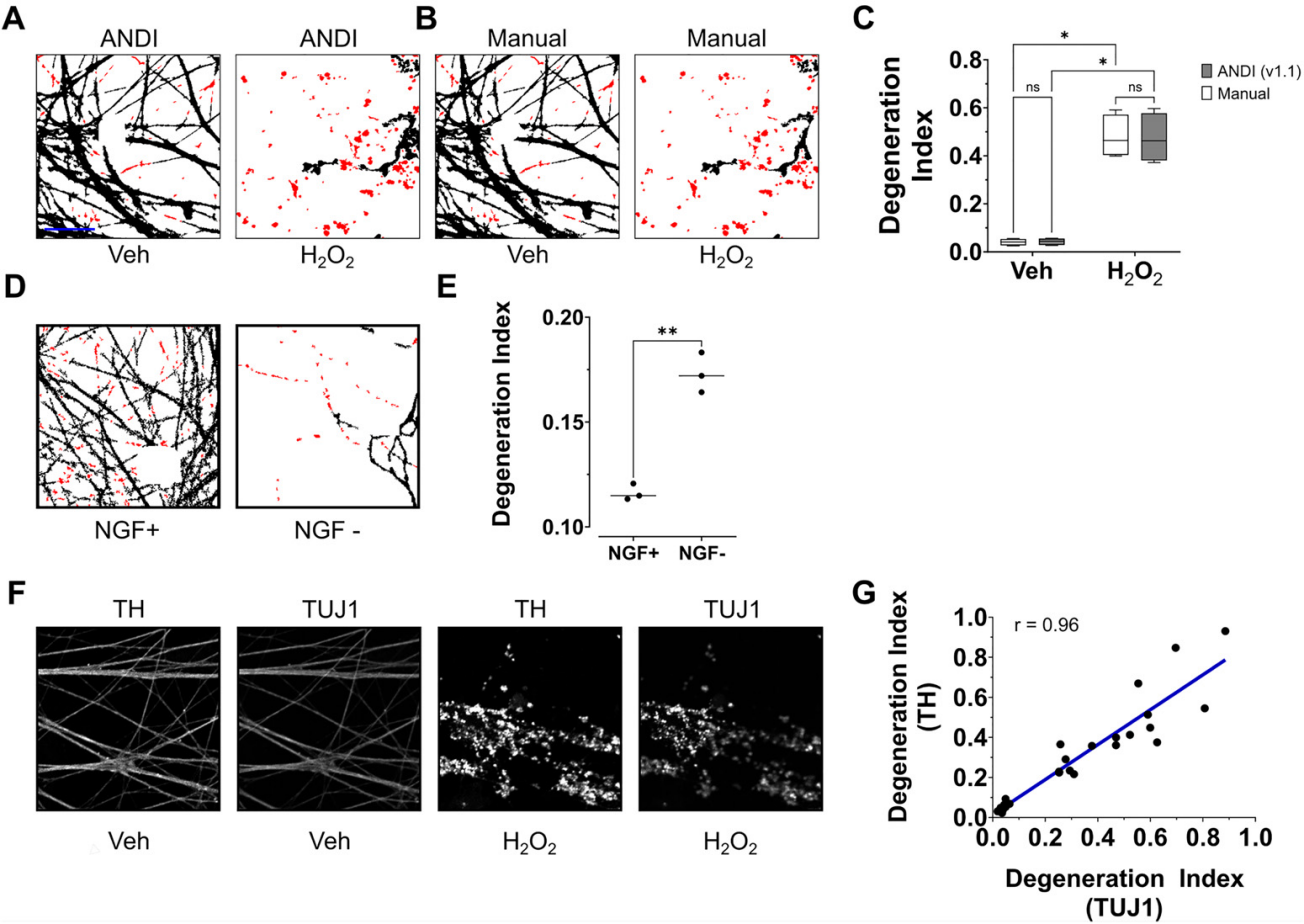
Utility of ANDI for measuring of neurite degeneration in primary cultures of sympathetic neurons. ***A***, ***B***, Representative images of cultured sympathetic neurons that were exposed to vehicle solution or 500 μm hydrogen peroxide for 24 h. After treatment, the neurons were fixed in 4% PFA, immunolabeled for βIII-tubulin, and counterstained with the nucleic acid dye DAPI. Images of the cells were then either subjected to automated soma removal, binarization, and neurite fragment analysis using ANDI (***A***) or were subjected to manual soma removal using the freehand drawing tool of ImageJ, followed by analysis of DI using parameters identical to those featured in ANDI (***B***). Detected neurite fragments are displayed in red. ***C***, DI scores associated with the experiments performed as described in ***A***, ***B***. DI scores from images of neurons exposed to hydrogen peroxide were significantly higher than those from images of neurons treated with vehicle solution (*n *=* *4, ANOVA with Šidák correction for multiple comparisons, *F*_(1,3)_ = 71.2, *p *=* *0.0035, *R*^2^ = 0.9301). No significant difference was found between DI scores obtained using ANDI and those obtained following manual soma removal (*n *=* *4 experiments, each featuring an independent cell culture preparation, ANOVA with Šidák correction for multiple comparisons, *F*_(1,3)_ = 0.1082, *p = *0.7639, *R*^2^ = 0.0000). ***D***, Representative images of healthy sympathetic neuron cultures (left) or sympathetic neuron cultures subjected to NGF withdrawal for 80 h (right). Fixed cells were immunolabeled for βIII-tubulin, counterstained with the nucleic acid dye Hoechst, and imaged by confocal microscopy. The displayed images were processed for automated soma removal, binarization, and fragment detection using ANDI. Neurite fragments are highlighted in red. ***E***, DI scores associated with the experiments described in ***D***. The DI scores of neurons subjected to NGF withdrawal were significantly higher compared with DI scores from healthy cultures (repeated measures ANOVA, *n* = 3 experiments, each featuring an independent cell culture preparation, *F*_(1,2)_ = 287, *p* = 0.0035, *R*^2^ = 0.958,). ***F***, Representative images of healthy, vehicle-treated sympathetic neuron cultures or degenerating sympathetic neuron cultures treated with 500 μm hydrogen peroxide. After a 24-h treatment period, the cells were fixed with 4% PFA in PBS and immunolabeled for TH or βIII-tubulin (TUJ1). Counterstaining was performed with the nucleic acid dye DAPI (data not shown). ***G***, Plot displaying the correlation between DI scores generated by ANDI from images depicting TH staining (*y*-axis) and from micrographs of βIII-tubulin staining (*x*-axis). Measurements were obtained from images of sympathetic neurons that were treated for 24 h with vehicle solution, 100 μm hydrogen peroxide, or 500 μm hydrogen peroxide, followed by fixation with 4% PFA, immunolabeling for TH and βIII-tubulin, and counterstaining with DAPI. A strong correlation was observed between DI scores from images of TH staining and DI scores from images of βIII-tubulin staining (Spearman’s ρ = 0.96, *n* = 30 images from 3 independent cell culture preparations). veh, vehicle; NGF+, incubated in media containing nerve growth factor; NGF–, incubated in media lacking nerve growth factor; *p < 0.05, **p < 0.01; *ns*, not significant. Scale bar: 25 μm.

After determining the accuracy and efficiency of the ANDI Macro for measuring neurite degeneration in differentiated LUHMES cells exposed to 6-OHDA, we evaluated the suitability of the macro for experiments involving other cell culture models. A pilot study was first conducted to assess the accuracy of ANDI in performing DI measurements using micrographs of primary sympathetic cultures immunolabeled for βIII-tubulin and DAPI. Interestingly, however, the cell bodies of sympathetic neurons are larger than those of differentiated LUHMES cells, and thus the operations in ANDI related to automated soma removal did not remove the entirety of the soma (data not shown). To overcome this issue, we revised ANDI to feature a dialogue box enabling users to choose the size of the soma removal by controlling the number of times that binarized micrographs of nuclei are dilated. This version of ANDI, version 1.1, was then used to perform DI measurements from micrographs depicting healthy, vehicle-treated sympathetic neurons or degenerating sympathetic neurons exposed to hydrogen peroxide. Images of neurons exposed to hydrogen peroxide yielded significantly higher DI scores compared with images of vehicle-treated neurons (*F*_(1,3)_ = 71.2, *p *=* *0.0035, *R*^2^ = 0.9301; Fig. 6*A*,*C*). Moreover, we compared these DI scores that were obtained using ANDI to DI measurements obtained from the same image set following manual removal of cell bodies from fluorescence micrographs using the freehand tool of ImageJ. There was no significant difference in DI scores calculated following manual soma removal compared with scores obtained using ANDI (*F*_(1,3)_ = 0.1082, *p *=* *0.7639, *R*^2^ = 0.0000; Fig. 6*A–C*). *Post hoc* tests also confirmed that DI scores obtained using ANDI were not significantly different both in the case of untreated neurons (*p *=* *0.0972) and in the case of neurons exposed to hydrogen peroxide (*p *=* *0.9986). Furthermore, DI scores produced from ANDI correlated nearly identically with DI scores obtained following manual removal of cell bodies from corresponding micrographs (*r*_(34)_ = 0.991, *p *<* *0.0001). These data demonstrate that ANDI is suitable for measuring neurite degeneration in cultures of primary neurons, with soma removal operations that the user can customize to match different neuron types. Additionally, the detection of neurite fragments in cultures exposed to hydrogen peroxide demonstrates the applicability of the macro for use in experiments involving neurite degeneration induced by sources other than 6-OHDA. To further verify the use of ANDI in detecting neurite degeneration associated with various causes, we also used the macro to measure neurite degeneration in micrographs of healthy sympathetic cultures or cultures subjected to NGF withdrawal. As expected, images of cultures subjected to NGF withdrawal yielded significantly higher DI values compared with images of healthy cultures (*F*_(1,2)_ = 287, *p *=* *0.0035, *R*^2^ = 0.958; Fig. 6*D*,*E*). Collectively, these findings demonstrate that ANDI can be used to detect neurite degeneration associated with a variety of biological contexts.

Since staining for TH is commonly used to visualize catecholinergic neurons, we also evaluated the suitability of micrographs depicting TH staining for measuring neurite degeneration using ANDI. Cultures of healthy sympathetic neurons treated with vehicle solution or degenerating sympathetic neurons exposed to hydrogen peroxide were fixed and subjected to immunofluorescence labeling for TH and βIII-tubulin, as well as counterstaining for DAPI. Neurite images depicting staining for TH or βIII-tubulin, and corresponding images of nuclei featuring DAPI labeling, were then captured at similar fields of view. ANDI was executed, and the images featuring TH staining or βIII-tubulin staining were selected as the neurite images. Our analyses revealed that DI scores obtained using images of TH staining correlated very strongly with scores obtained from images of βIII-tubulin staining (*r*_(28)_ = 0.962, *p *<* *0.0001; Fig. 6*F*,*G*). These data indicate that accurate DI measurements can be obtained by ANDI using alternative staining procedures that label the entirety of neurites, such as immunolabeling for TH.

**Figure 7.**
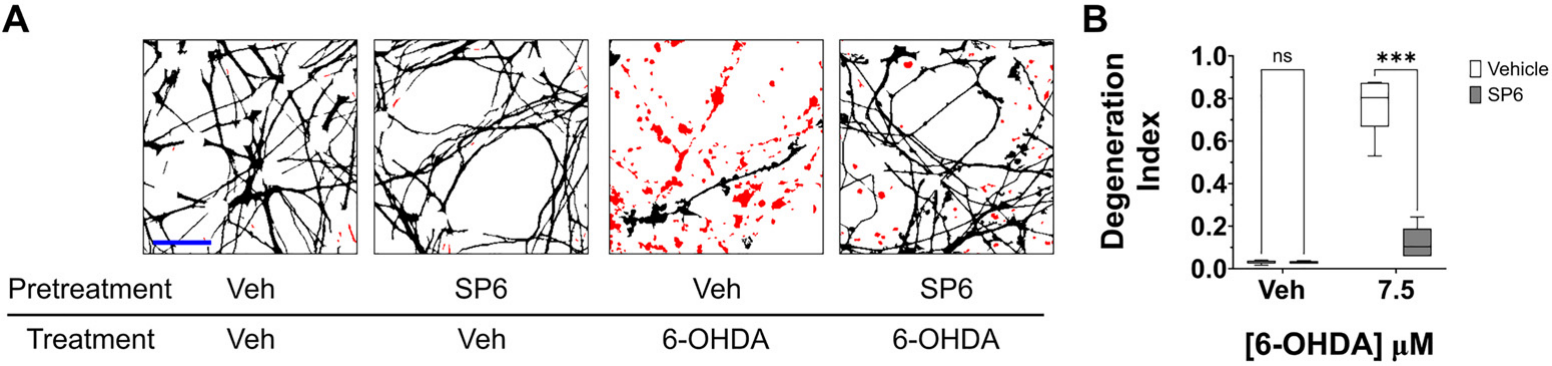
Use of the ANDI Macro to investigate the effects of JNK signaling on oxidative stress-induced neurite degeneration in human mesencephalic cells. ***A***, Representative images of differentiated LUHMES cells that were exposed for 24 h to vehicle solution or 7.5 μm 6-OHDA following 1-h pretreatment with 10 μm SP600125 or vehicle solution. Fixed cells were immunolabeled for βIII-tubulin and stained with DAPI, and fluorescence micrographs were analyzed using the ANDI Macro. Representative images that were processed for automated soma removal, binarization, and fragment detection are displayed. ***B***, DI scores associated with the experiments described in Figure 7*A*. Pretreatment with the JNK inhibitor SP600125 resulted in a significant decrease in neurite degeneration induced by 6-OHDA (*n* = 6, ANOVA with Šidák correction for multiple comparisons). veh, vehicle; SP6, SP600125; ***p < 0.001; *ns*, not significant; *n*, number of experiments, each featuring an independent cell culture preparation. Scale bar:* *25 μm.

To demonstrate the utility of the improved method for performing DI measurements in a scientific experiment, we used the new method to assess the role of JNK in oxidative stress-induced neurite degeneration in human mesencephalic cells. Differentiated LUHMES cells were pretreated for 1 h with the JNK inhibitor SP600125 or vehicle solution. The cells were then treated for 24 h with 6-OHDA to induce oxidative stress or with vehicle solution. Fixed cells were subjected to immunolabeling for βIII-tubulin and DAPI staining, and ANDI was used to obtain DI measurements from fluorescence micrographs. While cultures lacking pretreatment with the JNK inhibitor exhibited neurite degeneration following exposure to 6-OHDA, JNK inhibition resulted in a marked and significant reduction in 6-OHDA-induced neurite degeneration (*p *=* *0.0001, *d *=* *2.0209; Fig. 7). These findings support a key role for JNK signaling in neurite degeneration induced by oxidative stress in human mesencephalic cells, and importantly, demonstrate the utility of our new method for generating scientific discoveries by facilitating the rapid and accurate measurement of neurite degeneration.

## Discussion

Neurite degeneration is associated with a variety of neuropathological conditions, yet the molecular mechanisms underlying degradation of neurites remain incompletely understood. Methods facilitating accurate and efficient analysis of neurite degeneration are essential to the successful identification of novel factors regulating this important cellular event. In the present study, we reveal multiple sources of error associated with a commonly used method for quantifying neurite degeneration. We report experimental data supporting procedural modifications that can be implemented to reduce DI analysis error, and such modifications are incorporated into a new ImageJ macro to provide a tool for rapid, accurate, and objective DI analyses using open source and free software. Moreover, we demonstrate how the improved method can be applied to measure neurite degeneration in a cell culture model of PD, a disorder in which axonal fragmentation in dopaminergic neurons is an early-stage event that precedes eventual neuronal loss (Tagliaferro and Burke, 2016).

In the present study, we demonstrate the utility of the ANDI Macro in measuring neurite degeneration in a cell culture model of PD consisting of differentiated LUHMES cells exposed to 6-OHDA. We report experimental findings supporting the optimal substrates on which the cultures should be established, as well as demonstrate the plating density that is most appropriate for neurite degeneration analyses. Use of this model system to study neurite degeneration has several advantages. The cells develop neurites that are well-networked and typically >500 μm in length, and the cultures are susceptible to neurodegeneration induced by classic, neurotoxin models of PD such as 6-OHDA, 1-methyl-4-phenylpyridinium ion (MPP+), and rotenone (Lotharius et al., 2005; Zhang et al., 2014; Smirnova et al., 2016; Kraemer et al., 2021). Furthermore, the human origin of LUHMES cells increases the translatability of findings associated with the model, and the conditional immortalization facilitates rapid generation and propagation of cultures (Scholz et al., 2011). Thus, coupled with automated analyses performed using ANDI, LUHMES cells can be used to make novel discoveries related to PD-associated neurite degeneration while facilitating considerably higher throughput compared with nonautomated analyses performed with primary neuronal cultures.

Numerous research groups have performed DI analyses using phase-contrast images to gain insight into factors that regulate neurite degeneration (Press and Milbrandt, 2009; Kraemer et al., 2014; Di Stefano et al., 2015; Hill et al., 2018; Geisler et al., 2019). Here, we report an important drawback associated with this widely used method for measuring neurite degeneration: that binarization of phase-contrast images causes a significant proportion of intact neurites to appear fragmented. While the level of contrast that may be achieved when capturing micrographs varies depending on the microscope system available to investigators, this issue is apparently pervasive among various research groups, as a DI of 0.2 is commonly accepted as a threshold above which cultures are considered to have degenerating axons, and most scientific reports featuring DI measurements from phase-contrast micrographs have reported DI scores for healthy neurons between 0.1 and 0.3 (Sasaki et al., 2009, 2016; Di Stefano et al., 2015; Hill et al., 2018; Loreto et al., 2020). Our DI analyses obtained from fluorescence micrographs, as well as subjective scores from blinded investigators, indicate that 10–20% neurite fragmentation is not common to healthy neuron cultures. Rather, such cultures exhibit only 2.5% neurite fragmentation on average. Thus, our findings underscore the need for methods that improve the accuracy of neurite degeneration analyses by reducing this common and significant source of measurement error.

While the majority of studies involving DI measurements have used phase-contrast images to perform the neurite degeneration analyses, several research groups have recently performed DI measurements using images of neurons immunolabeled for cytoskeletal filaments such as neurofilament medium polypeptide or β-tubulin (Wakatsuki et al., 2011; Li et al., 2017; Pease-Raissi et al., 2017; Hernandez et al., 2018; Sundaramoorthy et al., 2020). However, which form of microscopy is best suited for neurite degeneration measurements has remained unclear, as investigations have been needed to understand whether changes in the localization of these cytoskeletal proteins during neurite degeneration appropriately model total changes in neurite morphology, as well as to evaluate the degree to which DI measurements obtained with fluorescence micrographs correlate with similar measurements obtained from phase-contrast micrographs. Here, we demonstrate that fluorescence micrographs depicting βIII-tubulin staining not only accurately represent neurite fragmentation, but also facilitate more accurate DI measurements because of their superior contrast and decreased susceptibility to artificial fragmentation on binarization. Thus, our results highlight the utility of using βIII-tubulin staining to measure neurite degeneration.

Among the published studies involving DI analyses to measure neurite degeneration, the reported size and circularity parameters used to detect neurite fragments have considerably varied. For example, a recent study involving assessment of neurite degeneration in cultured hippocampal neurons used size parameters of 4–900 pixels (Li et al., 2017), while a study involving cultured DRG neurons used detection parameters of 0–10,000 pixels (Wakatsuki et al., 2011). One potential reason for such variation is that the efficacy of particle analysis size parameters in detecting neurite fragments varies depending on the resolution of the image, since the size parameters for particle detection are associated with pixel units. Unfortunately, most published studies involving DI analyses do not include a description of the image resolution used for the analyses. Thus, the establishment of standard analysis parameters via a protocol that fully discloses important details such as the recommended image resolution has been needed. Here, we report that the most commonly-cited parameters for neurite fragment detection, 20–10,000 pixels (Shin et al., 2012; Di Stefano et al., 2015; Sasaki et al., 2016; Loreto et al., 2020; Shin and Cho, 2020), result in large proportions of neuron fragments being undetected. Reducing the minimum size criterion for neurite fragment detection enhances the sensitivity of fragment detection, while simultaneously increasing the number of image artifacts and non-neurite debris that are falsely detected as neurite fragments. In search for parameters that would best balance detection sensitivity and false-positivity rates, we identified analysis parameters of 10–10,000 as providing the most accurate detection of neurite fragments in binarized images obtained from phase-contrast micrographs, while parameters of 5–10,000 facilitate the most accurate detection of neurite fragments in binarized images obtained from fluorescence micrographs. While these determinations were made using an image resolution of 1280 × 1024, such parameters should also enable accurate neurite fragment detection in images captured at other resolutions featuring a vertical size of 1024 pixels, such as the common full screen resolution of 1024 × 1024, since neurons depicted in such images would be of similar pixel size.

Numerous research groups have used a minimum size criterion when configuring particle analysis parameters to avoid small, non-neurite debris from being included in neurite fragment measurements (Shin et al., 2012; Cosker et al., 2013; Di Stefano et al., 2015; Sasaki et al., 2016; Li et al., 2017; Loreto et al., 2020; Shin and Cho, 2020; Sundaramoorthy et al., 2020). However, such studies have erroneously included small, non-fragment matter in the measurements of total neurite area, since such measurements are obtained by summing the area of all black pixels in the image. Here, we demonstrate the utility of the Particle Remover plugin to remove small, non-fragment debris from the image before DI analysis. Utilization of the plugin significantly increased the sensitivity of neurite degeneration measurements obtained from phase-contrast images. Thus, our results highlight the value of the Particle Remover plugin for investigators performing DI analyses using phase-contrast micrographs. Interestingly, fluorescence micrographs featured less small, non-neurite debris, and thus, a lower size criterion of five pixels could be used for fragment detection. While particle removal of non-neurite debris less than five pixels in size did not significantly affect DI measurements obtained from fluorescence micrographs, such operations provide values that are theoretically more accurate and therefore were included in ANDI.

Since DI analyses must be performed using images exclusively featuring neurites, application of the analysis method has primarily been limited to use with culture systems amenable to removal of cell bodies, such as explant cultures in which cell bodies can be physically excised (Catenaccio et al., 2017; Shin and Cho, 2020) or cultures in microfluidic devices facilitating segregation of soma and axon compartments (Cosker et al., 2013; Pease-Raissi et al., 2017; Tan et al., 2018). Alternatively performing DI analyses with dissociated cultures can be achieved through tedious and time consuming labor associated with digital removal of cell bodies from micrographs (Kraemer et al., 2014). In the present study, we reveal that ANDI enables performance of DI measurements from micrographs featuring LUHMES cells that are dissociated and mosaically-distributed across a 2d culture system. Our findings also demonstrate that ANDI facilitates a 24-fold decrease in time required to perform the analyses, and the operations in ANDI related to soma removal yield DI analysis results that correlate 98% with similar analyses performed following manual soma removal using the freehand tool of ImageJ. Thus, the macro substantially increases the efficiency of the analyses by performing automated soma removal via a process that does not sacrifice accuracy, and such operations expand the suitability of the method for analyzing dissociated cultures with mosaically-distributed cell bodies.

In addition to facilitating automated cell body removal from images, ANDI features a several revisions to the traditional method for performing DI analysis, including use of fluorescence micrographs, revised particle analysis parameters, and utilization of the particle remover to remove small, non-neurite debris from images. Our findings indicate that these optimizations collectively yield DI values that more closely approximate values obtained through blinded and subjected scoring. Thus, in addition to substantially reducing the time required to perform the analyses, ANDI facilitates DI measurements that, compared with the traditional method, more accurately reflect the neurite degeneration that investigators reportedly observe from qualitative image analysis.

One drawback of the traditional method for performing DI analyses is that such analyses require training in use of ImageJ so that the user is familiar with a tedious set of ImageJ operations. Moreover, to perform the analyses in an efficient manner, further training is required for the user to perform batch analyses or to write scripts to semi-automate execution of particular steps. By fully automating the analysis, ANDI facilitates DI measurements in only a few steps related to downloading and executing the macro. To increase user friendliness, the macro includes code to provide an interface for selecting directories containing images to be analyzed, as well as to facilitate output of result images and data tables to a directory of the user’s choosing. Moreover, the script contains numerous features designed to prevent measurement inaccuracies or user mistakes, including automated clearing of previous ImageJ results; removal of image scale information to ensure measurements are accurately performed using pixel units; appropriate configuration of ImageJ color settings, measurement settings, and particle analyzer parameters; error message presentation if users erroneously select directories containing unequal numbers of images depicting βIII-tubulin staining and DAPI staining; log descriptions indicating analysis progress; and coding to prevent bugs associated with automated .ini file generation by Windows 10. Altogether, these features make DI analyses more user friendly, which may foster interest in studies related to neurite degeneration.

To demonstrate its utility in an experiment, we used ANDI to evaluate the contributions of JNK to neurite degeneration in LUHMES cell cultures exposed to 6-OHDA. JNK is a stress-activated kinase that has been reported as key to neurite degeneration in peripheral neurons of nonprimate origin (Shin et al., 2012), but further studies are needed to understand the role of the kinase in neurite degeneration in mesencephalic human cells. Here, we demonstrate that inhibition of JNK significantly protects human mesencephalic cells from neurite degeneration associated with oxidative stress. Such findings not only reveal an important role for JNK in neurite degeneration associated with a model of human disease, but also exemplify the utility of ANDI for performing automated and objective image analysis to make new scientific discoveries.

While ANDI was initially designed to facilitate rapid and accurate measurement of neurite degeneration in cultures of differentiated LUHMES cells exposed to 6-OHDA, our findings also indicate that the macro can be used to detect neurite degeneration induced by other causes, such as exposure to hydrogen peroxide or neurotrophin withdrawal. Additionally, version 1.1 of the macro features a dialogue box enabling investigators to customize the soma removal operations for neurons of specific sizes. Our data also supports that staining methods other than immunolabeling for βIII-tubulin can be used for accurate measurement of neurite degeneration. Although we recommend the use of pilot studies to verify the accuracy of DI measurements when using ANDI with cell types or staining methods that have not formerly been tested, these findings support that the macro is a generally versatile tool for measuring neurite degeneration in cultured neurons. Since ANDI has not been optimized for measuring neurite degeneration in tissue, we currently only recommend its use for analyzing neurite degeneration associated with cultured cells. However, the code for ANDI is publicly available and thus may also be useful for investigators interested in modifying the script to suit *in vivo* studies or other applications.

In conclusion, this article presents data revealing common sources of error associated with a widely used method for quantifying neurite degeneration. Experimental approaches were used to improve the method, and such enhancements were incorporated into a free and open-source ImageJ macro that can be used to perform rapid, accurate, and objective analysis of fluorescence micrographs for neurite degeneration. Our findings reveal the efficacy of ANDI for quantifying neurite degeneration in micrographs depicting a cell culture model of PD, as well as provide proof of principle that the macro can detect neurite degeneration associated with other biological contexts. The user-friendly macro will reduce the time required for investigators to learn to quantify neurite fragmentation, while increasing the throughput and accuracy of studies evaluating factors underlying neurite degeneration. The ANDI Macro is available at the following URL: https://github.com/kraemerb/kraemerlab.

## Synthesis

Reviewing Editor: Eran Perlson, Tel Aviv University Decisions are customarily a result of the Reviewing Editor and the peer reviewers coming together and discussing their recommendations until a consensus is reached. When revisions are invited, a fact-based synthesis statement explaining their decision and outlining what is needed to prepare a revision will be listed below. The following reviewer(s) agreed to reveal their identity: Laura Volpicelli-Daley, Gerard O’Keeffe.

The concept and centrality of axonal degeneration as an early pathophysiological process in Parkinson’s disease has emerged in recent years. The development therefore of tools to analyzed and improve the study on axonal degeneration is timely and will be a significant addition to many labs in the field. The authors provide an outstanding description of their methods, statistics and the images provided are of high quality. It is really nice that the authors are analyzing the neurodegeneration process- differentiated cells are treated with 6-OHDA and degeneration is analyzed; and not just measure neurite outgrowth which may not be relevant for neurodegenerative disease studies. All in all, this is a fine study.

Although important to the field the authors should provide some clarification:

1. The LUHMES cells are used in PD research, but are not necessarily the most common cell culture model. While I understand the rational of using LUMHES cells in that they are convenient to work with and extend extensive axons, have the authors also tried this method in mixed cultures et E14VM and specifically on cultures stained for TH? It would be extremely helpful to know how generalizable this method is to other cell types such as differentiated iPSCs or primary neurons. The BetaIII-tubulin immunofluorescence may not appear as continuous in these other cells. 6-OHDA may not induce neurodegeneration in the other cell types- H2O2 or even colchicine may work as proof of principle. The concentrations of 6OHDA used in the current study induce axon degeneration in primary cultures of the E14 rat VM after 72h in vitro. As these DA neurons in these cultures represent about 10% of the population, does this mention also work in this setting?

2. While I understand the authors are focusing on the method and using 6-OHDA as an exemplar of neurite degeneration, so the authors expect this approach would also work in cells over expressing aSyn?

3. Having the authors tried the approach in vivo? 6-OHDA and indeed aSyn induces axonal blebbing and fragmentation in the striatum at high power magnificent. I am not suggestion the authors perform an in vivo experiment but rather if they had access to a data set of images perhaps the approach could be tested on these? Or discuss in the discussion section.

4. Figure 1: legend for B says order of images are: 200 μg/mL poly-

800 L-ornithine and 2 μg/mL fibronectin, 100 ng/mL poly-D-lysine and 10 μg/mL laminin, all four substrates, or no 801 substrates. But in the figure the images are ordered by all 4 substrates, PDL+laminin, PLO + Fibro or no. To make it easier to follow, I recommend re-organizing the list so it is consistent with the figure.

5. Can the authors please provide DAPI images and examples of how live vs. dying cells were scored?

6. In figure 6, should the 3rd panel be VEh/6-OHDA instead of SP6/6-OHDA

7. It would be VERY helpful to have some FIJI and Image J screen shots of the commands used and how to find them.

## Author Response

MANUSCRIPT NUMBER: eN-MNT-0327-21

TITLE: Quantification of Neurite Degeneration with Enhanced Accuracy and Efficiency in an In Vitro Model of Parkinson’s disease.”

Response to Reviewer Comments:

The authors would like to thank the reviewers and editor for recognizing the importance of this research and for providing insightful comments about the manuscript. We have carefully considered the reviews and have addressed the concerns as detailed in the point-by-point response below. We appreciate the effort that was dedicated to providing us with helpful feedback and to facilitating improvement of the manuscript.

Clarification #1

The LUHMES cells are used in PD research, but are not necessarily the most common cell culture model.

While I understand the rational of using LUMHES cells in that they are convenient to work with and extend extensive axons, have the authors also tried this method in mixed cultures et E14VM and specifically on cultures stained for TH? It would be extremely helpful to know how generalizable this method is to other cell types such as differentiated iPSCs or primary neurons. The BetaIII-tubulin immunofluorescence may not appear as continuous in these other cells. 6-OHDA may not induce neurodegeneration in the other cell types- H2O2 or even colchicine may work as proof of principle. The concentrations of 6OHDA used in the current study induce axon degeneration in primary cultures of the E14 rat VM after 72h in vitro. As these DA neurons in these cultures represent about 10% of the population, does this mention also work in this setting?

We agree that it would be beneficial to demonstrate that this new method is generalizable to experiments involving other cell types, including primary neurons. Although our laboratory does not have expertise in generating ventral midbrain cultures, we have now added to the manuscript data demonstrating use of ANDI to measure neurite degeneration in primary cultures of sympathetic neurons. Like dopaminergic neurons from the ventral midbrain, sympathetic neurons are catecholinergic neurons that are susceptible to Lewy pathology and neurodegeneration associated with early stages of Parkinson’s disease1-3. Interestingly, the cell bodies of sympathetic neurons appeared to be larger than those of differentiated LUHMES cells, and thus, during our initial attempts to measure neurite degeneration from micrographs of sympathetic neurons, the commands in ANDI related to automated soma removal did not remove the entirety of the soma. To overcome this issue, we revised ANDI (termed ANDI version 1.1) to include a dialog box that allows users to choose the size of the soma removal (by controlling the number of times the binarized micrographs of nuclei are dilated). This modification not only enabled accurate soma removal in micrographs of sympathetic neurons, but also generally improved the user friendliness and applicability of the macro by providing users with a straightforward way to customize the soma removal operations to suit different cell sizes.

We appreciate the suggestion to use a different toxin, such as H2O2 or colchicine, to provide proof of principle that the ANDI macro can measure neurite degeneration induced by different causes. The revised manuscript includes new data demonstrating the ability of the ANDI to detect neurite degeneration in primary cultures of sympathetic neurons exposed to H2O2. These data also reveal that ßIII tubulin is continuous in healthy sympathetic neurons and can be used to detect neurite fragments even at severe stages of neurite degeneration (Fig. 6A). Although the cultures of sympathetic ganglia were treated with cytosine arabinoside to limit growth of non-neuronal cells, the cultures still retained a variety of Schwann cells and fibroblasts. However, ßIII-tubulin is exclusively expressed in neurons, and thus, despite the presence of the non-neuronal cells, the DI scores obtained from images of healthy neurons and degenerating neurons were in the ranges expected (Fig. 6C). Since sympathetic neurons express tyrosine hydroxylase (TH), we also performed degeneration index measurements using micrographs depicting TH immunolabeling. Data has been added to the revised manuscript revealing a high correlation between DI scores obtained using micrographs depicting TH-immunoreactivity and DI scores from micrographs depicting ßIII-tubulin immunoreactivity (Fig. 6F-G). While we recommend the use of pilot studies verify the accuracy of DI measurements when applying ANDI to analyze untested cell types, these findings collectively provide proof of principle that ANDI can accurately measure neurite degeneration in cultures featuring a variety of cell types when a stain exclusive to the cell type of interest, such as immunolabeling for TH or ßIII-tubulin, is applied. Discussion pertaining to the application of ANDI to measure neurite degeneration in different types of cultures has been added to the revised manuscript.

Clarification #2

While I understand the authors are focusing on the method and using 6-OHDA as an exemplar of neurite degeneration, so the authors expect this approach would also work in cells over expressing aSyn?

Yes, the operations in ANDI detect neurite fragments based on size and circularity of image objects, and thus this approach is expected to accurately measure neurite degeneration induced by any injury or condition that produces small neurite fragments. One general limitation of DI measurements - including those obtained using traditional methods or ANDI - is that the method has low sensitivity for early stages of neurite degeneration in which neurite beading occurs but fragments have not yet detached from the axon. However, DI analyses are suitable for conditions associated with moderate or severe neurite degeneration in which neurite fragmentation or detachment of matter from the neurite occurs. From review of published articles featuring micrographs of neurons overexpressing α-synuclein, it appears that α-synuclein overexpression increases neurite fragmentation.4, 5 Thus, we expect that ANDI could be used to measure this form of neurite degeneration. The addition to the manuscript of data demonstrating use of ANDI to measure neurite degeneration induced by H2O2 exemplifies the utility of the macro for measuring neurite degeneration induced by factors other than 6 OHDA. We also recently received from an investigator of a different institution images of healthy cultures of sympathetic neurons and sympathetic neurons subjected to neurotrophin withdrawal. Although the images that we received had low contrast, thus resulting in slightly higher DI values for the healthy neurons, the ANDI macro successfully detected a significant increase in neurite degeneration induced by neurotrophin withdrawal. These data have been added to the manuscript to provide additional evidence of the utility of the macro for measuring neurite degeneration associated with a variety of causes (Fig. 6D-E).

Clarification #3

Having the authors tried the approach in vivo? 6-OHDA and indeed aSyn induces axonal blebbing and fragmentation in the striatum at high power magnificent. I am not suggestion the authors perform an in vivo experiment but rather if they had access to a data set of images perhaps the approach could be tested on these? Or discuss in the discussion section.

This is an interesting idea. To explore this possibility, we performed a pilot experiment in which a C57Bl6 mouse was administered 10 mg/kg desipramine (to protect noradrenergic terminals from 6-OHDA exposure) 30 minutes prior to unilateral, intrastriatal administration of 8.75 μg 6 OHDA at the following stereotaxic coordinates from bregma (+0.5 mm anterior - posterior, -1.9 mm medial-lateral, +3.5 mm dorsal-ventral). We collected and fixed the brain tissue three weeks post-treatment, and striatal cryosections were subjected to immunofluorescence staining for tyrosine hydroxylase (Sigma Cat# AB152) and imaged at 63X magnification using a Zeiss LSM 800 confocal microscope. ANDI was then used to analyze micrographs depicting the uninjected striatal hemisphere or the striatal hemisphere administered 6 OHDA. Although images from the hemisphere treated with 6-OHDA yielded higher DI scores, the micrographs of the uninjected hemisphere had impractically high DI values (Injected hemisphere, DI = 0.73, Uninjected hemisphere, DI = 0.41, mean of six micrographs per hemisphere).

Based on these preliminary findings, we think that ANDI could be feasibly modified to accurately measure neurite degeneration in vivo; however, such modifications would require extensive experimentation and revision of the macro to a degree beyond the scope of the current research. Additional studies would be needed to optimize the particle analysis parameters for accurate detection of fragments at 63X magnification, and strategies would likely need to be developed to minimize false positives associated with intact neurites that project in a direction orthogonal to the plane of focus. Based on these observations, we have added discussion to the manuscript indicating that ANDI is designed for measuring neurite degeneration in vitro, while also noting that the publicly accessible code in the macro may be useful for investigators wishing to modify the script to suit additional applications.

Clarification #4

Figure 1: legend for B says order of images are: 200 μg/mL poly-L-ornithine and 2 μg/mL fibronectin, 100 ng/mL poly-D-lysine and 10 μg/mL laminin, all four substrates, or no 801 substrates. But in the figure the images are ordered by all 4 substrates, PDL+laminin, PLO + Fibro or no. To make it easier to follow, I recommend re-organizing the list so it is consistent with the figure.

We appreciate this suggestion and have revised the manuscript such that the results section, legend for Figure 1, and Figure 1 images discuss or present the treatment conditions in the same order: no substrates, 200 μg/mL poly-L-ornithine and 2 μg/mL fibronectin, 100 ng/mL poly-D-lysine and 10 μg/mL laminin, or all four substrates.

Clarification #5

Can the authors please provide DAPI images and examples of how live vs. dying cells were scored?

We have now added to Figure 1 representative images depicting DAPI staining. We have also included in the figure arrowheads to exemplify DAPI-stained nuclei that were excluded from live cell counts due to low nuclear area and/or fragmentation indicative of apoptosis.

Clarification #6

In figure 6, should the 3rd panel be VEh/6-OHDA instead of SP6/6-OHDA

We appreciate the reviewers’ identification of this error, as the second and third images of Fig. 6A were indeed mislabeled. Figure 6 of the revised manuscript has been modified to correct this error. Thank you for facilitating this correction.

Clarification #7

It would be VERY helpful to have some FIJI and Image J screen shots of the commands used and how to find them.

Thank you for this recommendation. The document titled “Instructions.docx” in the ANDI.zip file that was included as multimedia/extended data has now been revised to feature images depicting each step needed to install and use ANDI. We have also added to that document a copy of the operations in ANDI and a list of strategies for troubleshooting common mistakes. We hope that this supporting documentation will be useful for members of the scientific community interested in utilizing this improved and automated method for investigating neurite degeneration. We appreciate this suggestion, as well as your other recommendations that have enabled improvements to the manuscript.

References

1. Del Tredici, K., Hawkes, C.H., Ghebremedhin, E. & Braak, H. Lewy pathology in the submandibular gland of individuals with incidental Lewy body disease and sporadic Parkinson’s disease. Acta Neuropathol 119, 703-713 (2010).

2. Orimo, S. et al. Axonal alpha-synuclein aggregates herald centripetal degeneration of cardiac sympathetic nerve in Parkinson’s disease. Brain 131, 642-650 (2008).

3. Bloch, A., Probst, A., Bissig, H., Adams, H. & Tolnay, M. Alpha-synuclein pathology of the spinal and peripheral autonomic nervous system in neurologically unimpaired elderly subjects. Neuropathol Appl Neurobiol 32, 284-295 (2006).

4. O’Donnell, K.C. et al. Axon degeneration and PGC-1alpha-mediated protection in a zebrafish model of alpha-synuclein toxicity. Dis Model Mech 7, 571-582 (2014). PMC4007408

5. Koch, J.C. et al. Alpha-Synuclein affects neurite morphology, autophagy, vesicle transport and axonal degeneration in CNS neurons. Cell Death Dis 6, e1811 (2015). PMC4650722
