# Supplementary material for: Quantification of Neurite Degeneration with Enhanced Accuracy and Efficiency in an In Vitro Model of Parkinson’s Disease
Source: eNeuro. 2022 Mar 17;9(2):ENEURO.0327-21.2022. doi: 10.1523/ENEURO.0327-21.2022 (PMC8938979; doi:10.1523/ENEURO.0327-21.2022)
Supplement: Extended Data 1 — ANDI v1.1 script for image processing and DI analysis using ImageJ. Download Extended Data 1, ZIP file. [file enu-eN-MNT-0327-21-s02.zip › ANDI_Instructions.docx]

**Instructions for Using ANDI**

*Please note that the ANDI macro has only been tested for use with a PC running Windows 10.*

Downloading Needed Files

1. Download the FIJI package containing ImageJ using the following url: <https://imagej.net/software/fiji/> . Extract the files to the directory of your choosing.


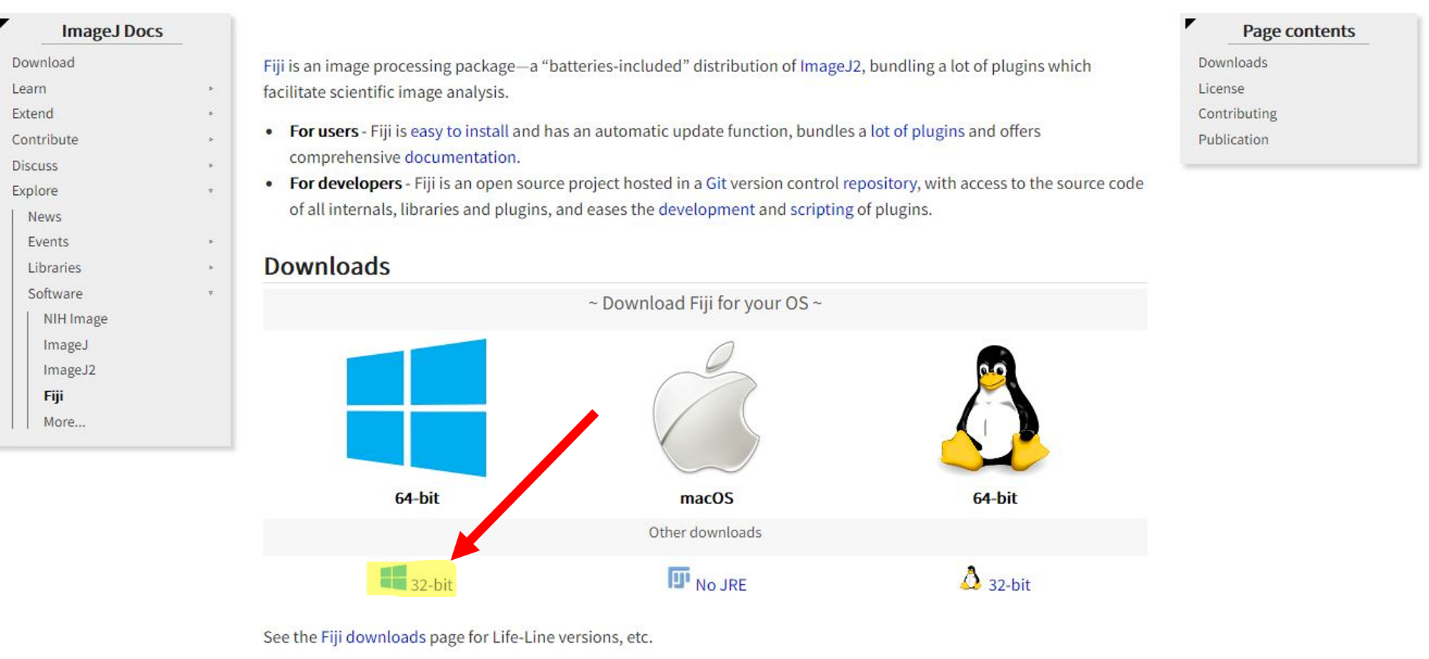


1. Visit <https://github.com/kraemerb/kraemerlab>
   - Click the green “Code” button and then click to download the .zip file. Extract the contents to the directory of your choosing.


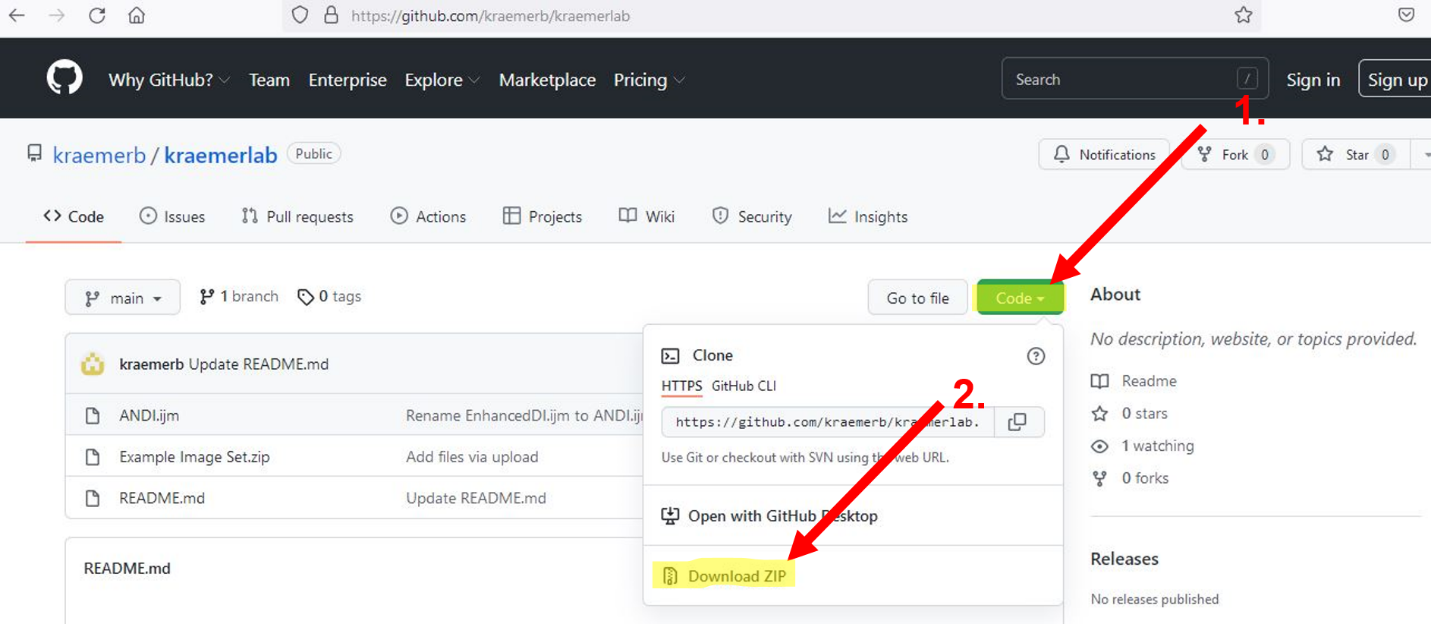


- - The contents of the zip file include:
    1. The ANDI macro (formatted as an .ijm file)
    2. An example image set
    3. Instructions for using ANDI

1. Download the two files associated with the Particle Remover plugin using the following url: <https://imagej.nih.gov/ij/plugins/particle-remover.html>


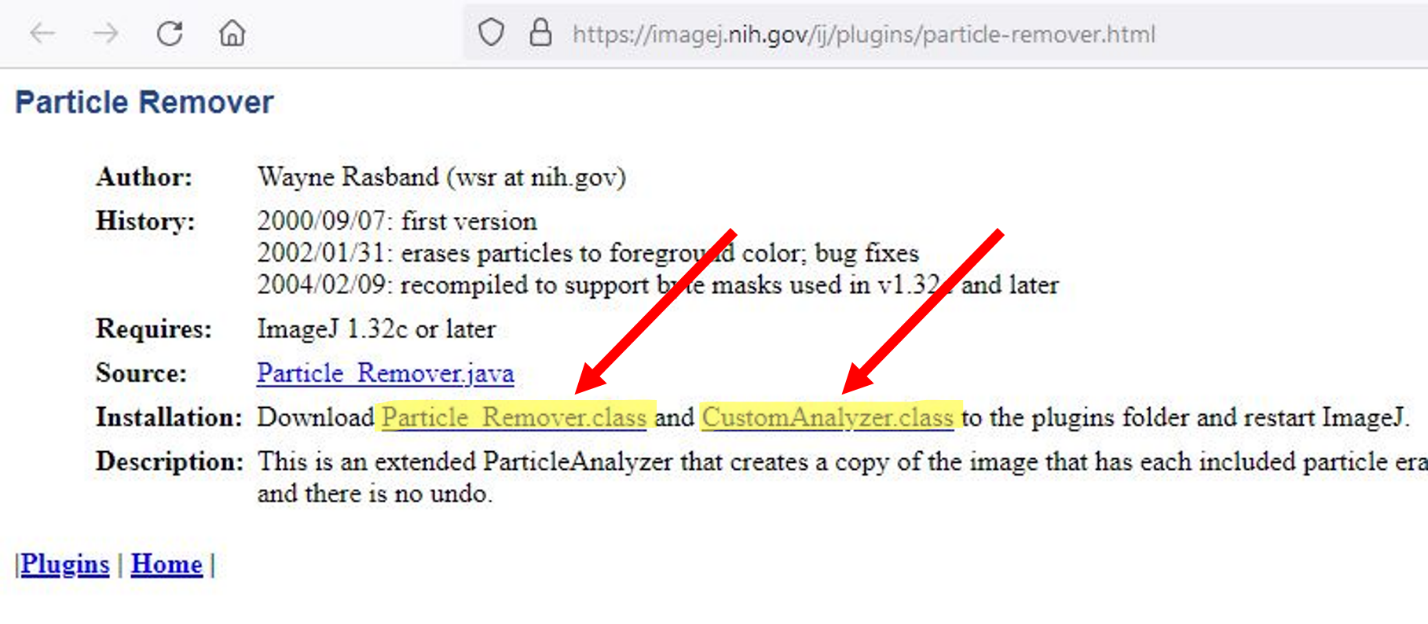


- - Place both files, Particle_Remover.class and CustomAnalyzer.class, in the Plugins folder of the directory in which ImageJ is installed.


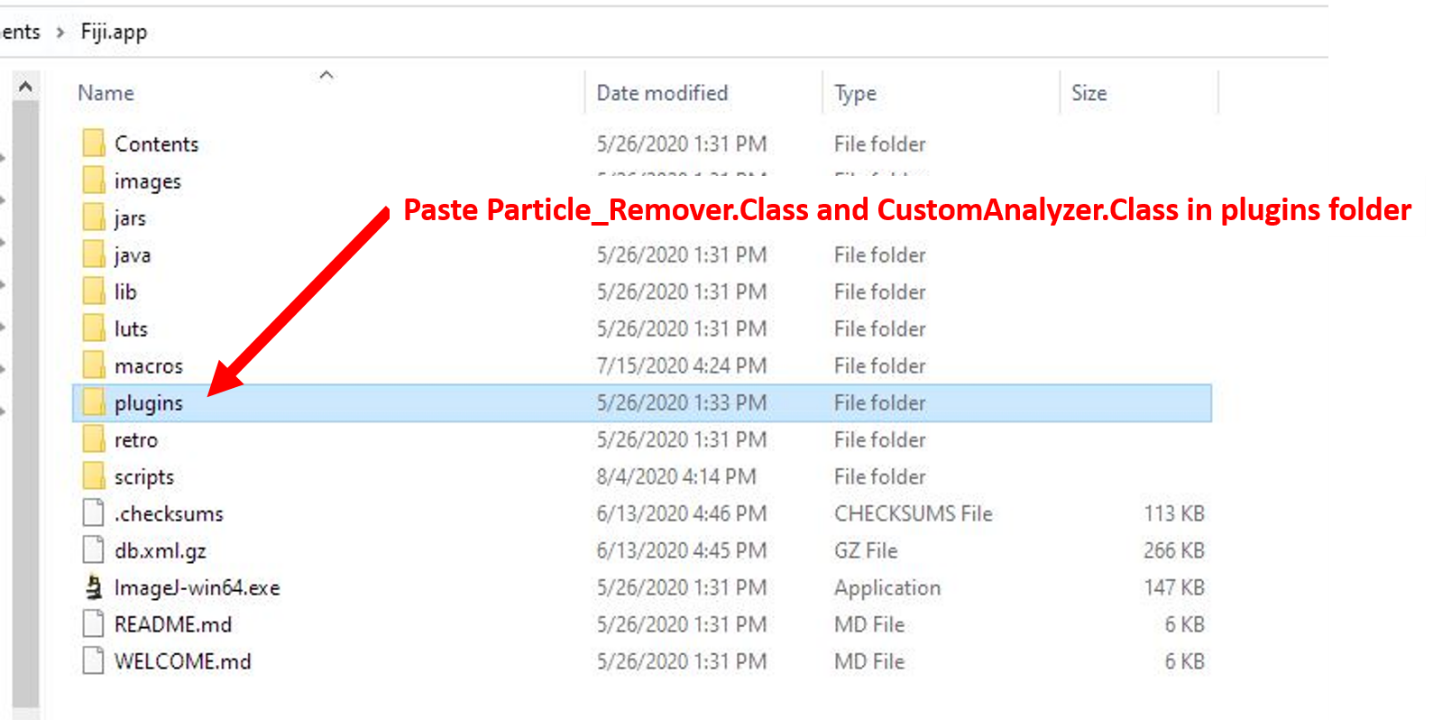


Preparation of Images

1. Use a 20X objective to capture fluorescence images featuring neurons (ie. βIII-tubulin staining) and corresponding images from the same field of view featuring nuclear staining (ie. DAPI staining).
   - Capture the image using a resolution with a vertical size of 1024 (ie. 1280 x 1024 or 1024 x 1024). If this cannot be done, software can be used to convert the image to such resolution, but the aspect ratio of the image must be maintained.


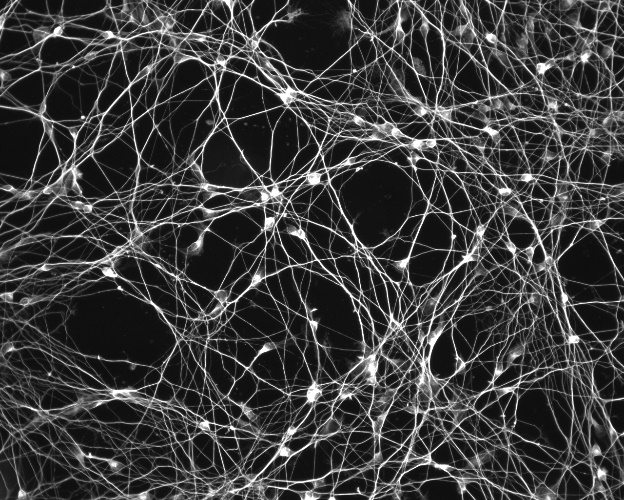


(Example Images)

1. Save all neuron images in one directory and all images of nuclear staining in a separate directory.
   - Ensure that no other files or folders are in the directories containing the images
   - Name the image files such that, within each directory, the images of nuclear staining are listed in the same order as the corresponding neuron images. The following are example file names that would appropriately list the neuron images and nuclei images in the same order:
     1. Example files names for directory containing neuron images:
        1. 062921_Tubulin_Well1_Image1
        2. 070321_Tubulin_Well2_Image1
        3. 070321_Tubulin_Well2_Image2
     2. Example file names for directory containing images of nuclear staining:
        1. 062921_DAPI_Well1_Image1
        2. 070321_DAPI_Well2_Image1
        3. 070321_DAPI_Well2_Image2


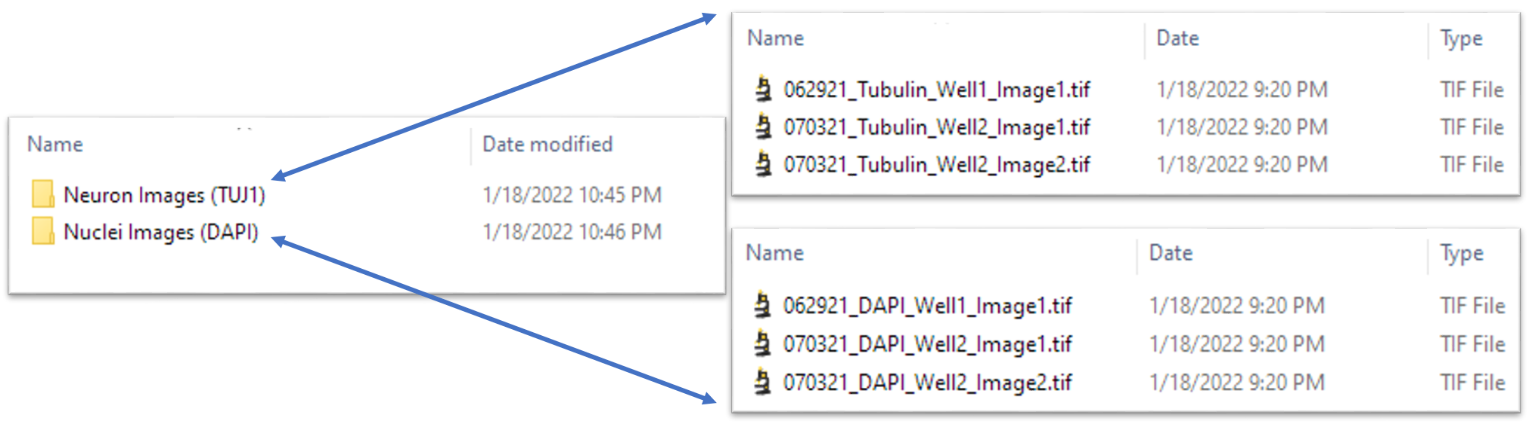


Executing the Macro

1. Open FIJI.
2. Click Plugins🡪Macros🡪 Run


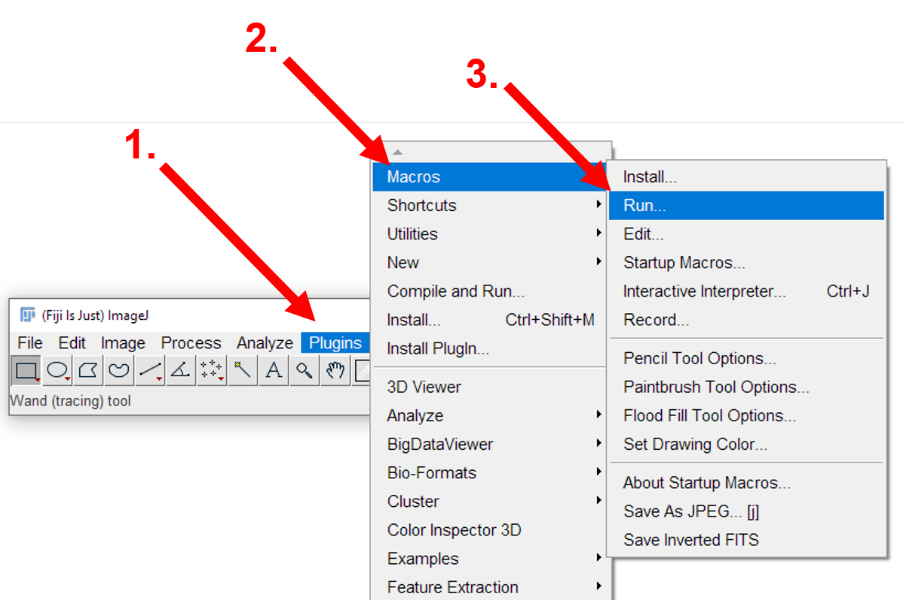


1. Select the ANDI.ijm file that you previously saved to a directory of your choosing, and click “open”


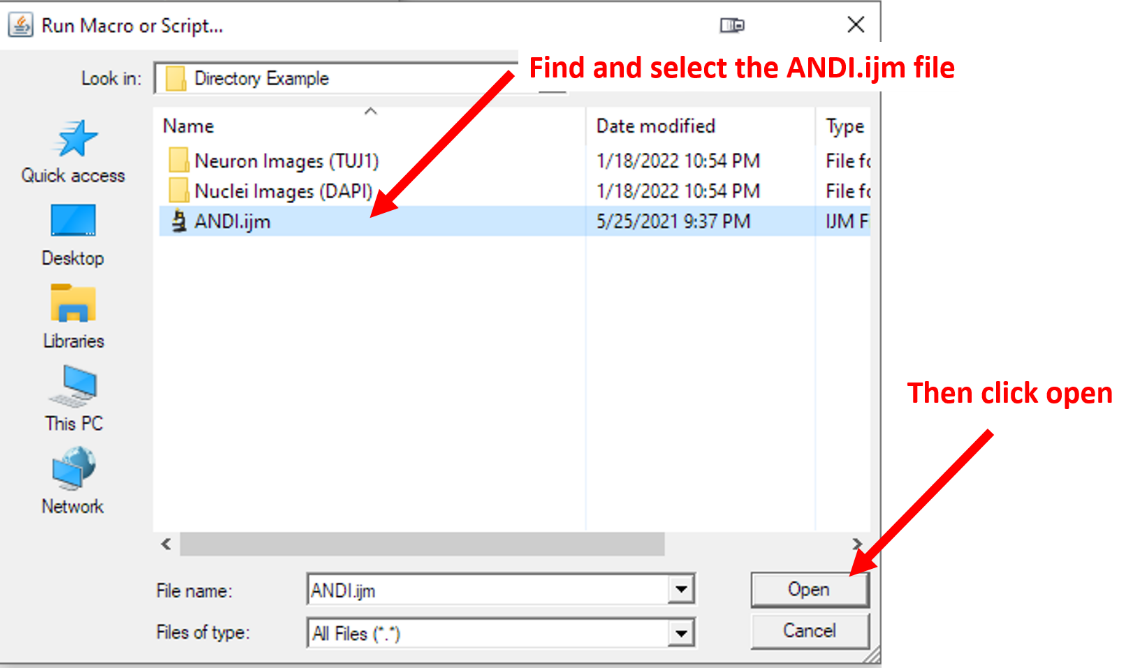


1. Follow the prompts to choose the directory containing the neuron images and the directory containing the nuclear staining images.


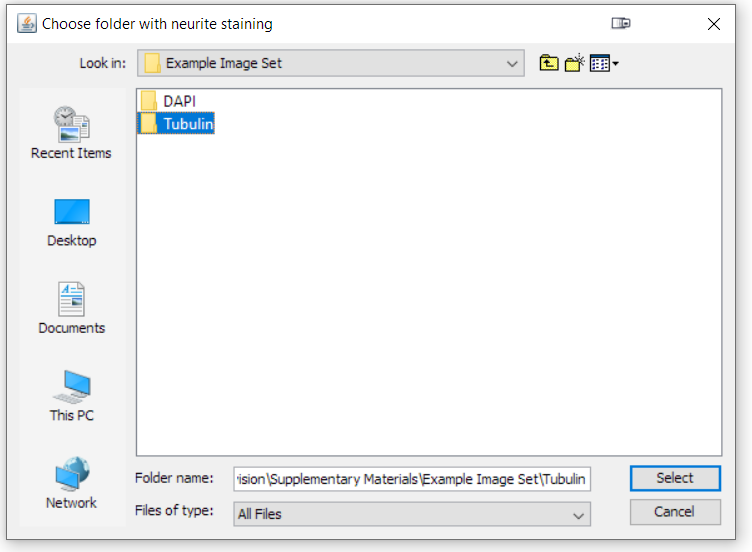


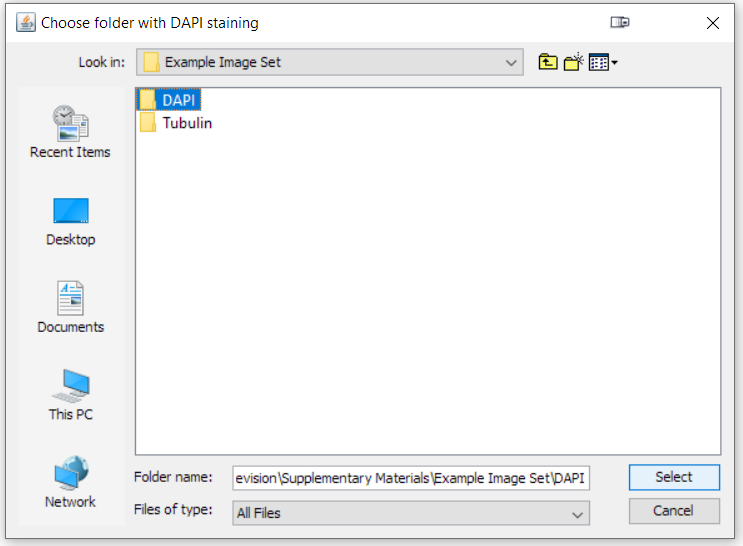


1. Choose the folder in which you would like the result files to be saved.


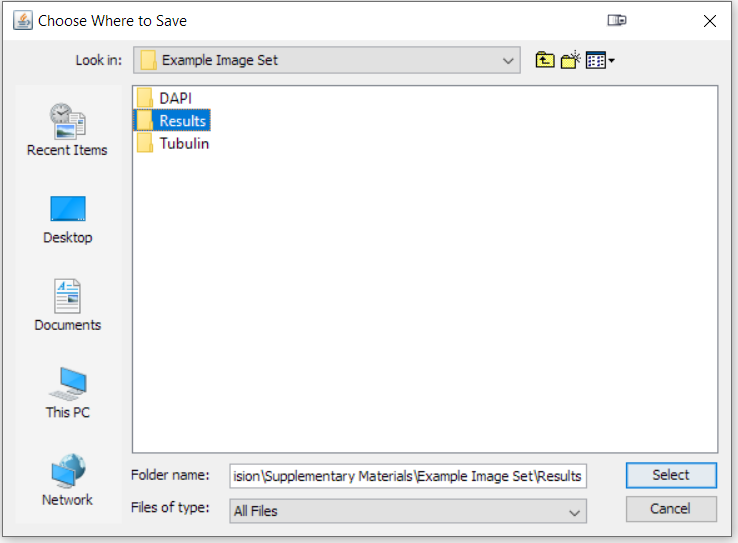


1. Type the number of times you would like the binarized nuclei to be dilated. Large numbers result in large soma removal. The recommended value for LUHMES cells is 12.


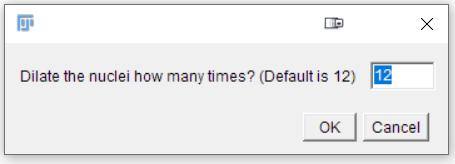


1. Do not move your mouse or operate other software while the macro executes its operations. When all calculations are complete, the log will display “Results have been saved in the selected directory.”

(Image on next page)


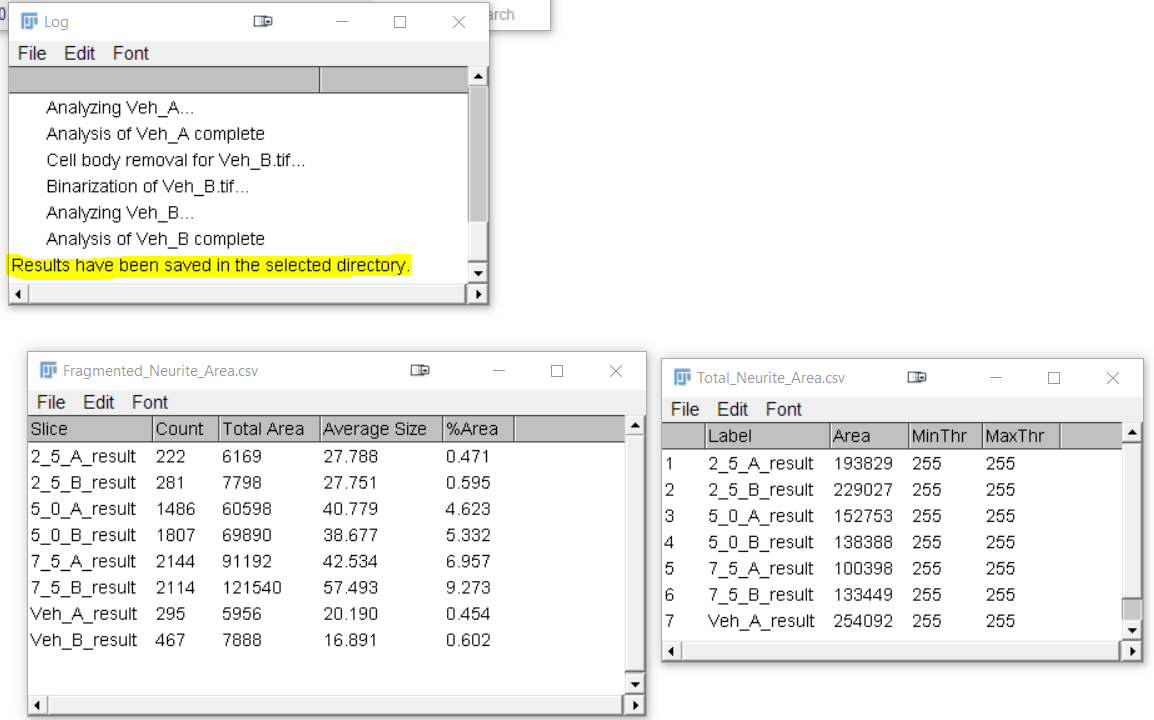


Calculating the Degeneration Index from the Result Files

1. Create a blank excel file with the title of your choosing (ie. “DI.xlsx)


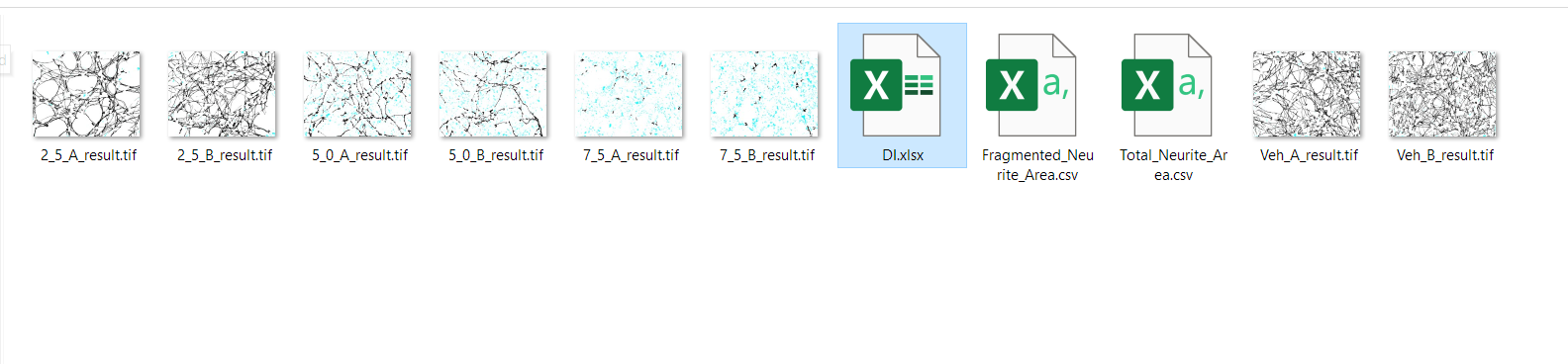


1. Open the result file titled Fragmented_Neurite_Area.csv and the result file titled Total_Neurite_Area.csv.
2. Paste the results from the two files in step 2 into the single excel file that you created in step 1.


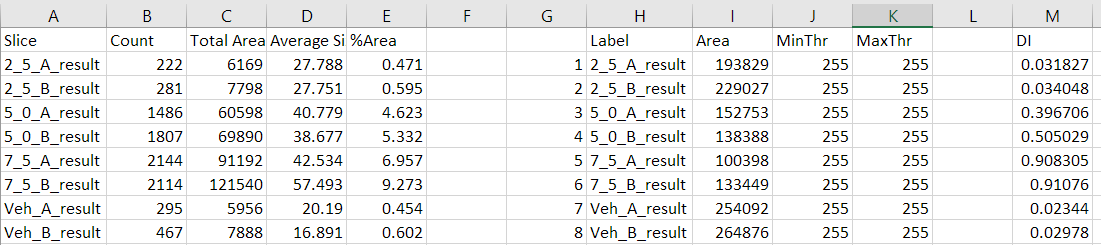


1. Divide the values in column C (highlighted in yellow below) of Fragmented_Neurite_Area.CSV by the corresponding values in column C of the Total_Neurite_Area.csv (displayed in column I and highlighted in blue after being pasted into DI. Xlsx) to obtain the Degeneration Index values of all images (highlighted in green).


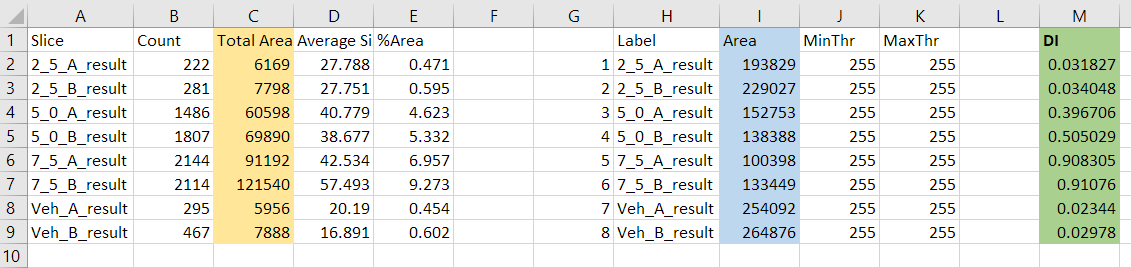


**Troubleshooting Tips**

| **Observation from Result Image** | **Cause** | **Solution** |
| --- | --- | --- |
| Result image is blank or has only a few image objects | DAPI image has poor contrast | When capturing DAPI images, increase camera exposure time, increase detector gain, or increase light intensity to generate a brighter DAPI signal. Alternatively, enhance the contrast of the DAPI images prior to analyzing the images with ANDI |
| Result image features small black pixels scattered throughout the image | Neurite image has poor contrast. | When capturing Neurite images, increase camera exposure time, increase detector gain, or increase light intensity to generate a brighter ßIII-tubulin signal. Alternatively, enhance the contrast of the neurite images prior to analyzing the images with ANDI |
| The binarization appears to be reversed (background is black and neurites are white) | Cell cultures are too dense. | Perform analyses with images featuring cells that are more sparsely distributed. Alternatively, manually binarize the neurite image using ImageJ prior to analyzing the images with ANDI. |
| Error message indicated that a window was not found | A program or activity disrupted the activity of the macro | Close background programs and avoid moving or clicking the mouse while executing the macro |
| Images of healthy neurons appear fragmented after binarization | Neurite image has poor contrast. | When capturing Neurite images, increase camera exposure time, increase detector gain, or increase light intensity to generate a brighter DAPI signal. Alternatively, enhance the contrast of the DAPI images prior to analyzing the images with ANDI |
| Cell bodies were not completely removed in the result images | Nuclei were dilated an insufficient number of times. | When executing ANDI, use the dialog box to increase the number of times that nuclei are dilated (default value is 12). |

**List of Operations Included in ANDI (v 1.1)**

setBatchMode(true);

//identify directories and create file lists

NeuriteDir = getDirectory("Choose folder with neurite staining");

DapiDir = getDirectory("Choose folder with DAPI staining")

SaveDir = getDirectory("Choose Where to Save");

NeuriteList = getFileList(NeuriteDir);

DapiList = getFileList(DapiDir);

if (NeuriteList.length != DapiList.length) {exit("number of images in two directories is not equal")};

p = getNumber("Dilate the nuclei how many times? (Default is 12)", 12);

run("Clear Results");

close("*");

//initiate for loop

for (f=0; f<NeuriteList.length; f++){

//code to prevent errors due to Desktop.ini files

IniFileIndex = indexOf(DapiDir + DapiList[f],".ini");

if(IniFileIndex >= 0) {

print("skipped due to .ini file detection");

continue;}

open(DapiDir + DapiList[f]);

OriDapiImage = getTitle();

run("8-bit");

run("Set Scale...", "distance=0 known=0 pixel=1 unit=pixel");

//run("Brightness/Contrast...");

setMinAndMax(10, 245);

run("Apply LUT");

//make binary and remove particles

run("Colors...", "foreground=white background=black selection=yellow");

setOption("BlackBackground", false);

run("Options...", "iterations=1 count=1");

run("Make Binary");

run("Particle Remover", "size=0-10");

close(OriDapiImage);

//dilate and erode

setOption("BlackBackground", false);

for (d=0; d<p; d++){

run("Dilate");

}

run("Erode");

run("Erode");

run("Erode");

run("Erode");

run("Erode");

rename("Y"+f);

//open neurite image

open(NeuriteDir + NeuriteList[f]);

OriNeuriteImage = getTitle();

//convert image to 8-bit and remove any scale information

run("8-bit");

run("Set Scale...", "distance=0 known=0 pixel=1 unit=pixel");

//contrast enhancement

setMinAndMax(0, 195);

run("Apply LUT");

print(" Cell body removal for " + OriNeuriteImage + "...");

//image subtraction

imageCalculator("Subtract create", OriNeuriteImage,"Y"+f);

//close old images and temporarily rename image with cell bodies removed

close(OriNeuriteImage);

close("Y" + f);

rename("X" + f);

print(" Binarization of " + OriNeuriteImage + "...");

//make binary and remove particles

setOption("BlackBackground", false);

run("Options...", "iterations=1 count=1");

run("Make Binary");

run("Particle Remover", "size=0-4");

close("X" + f);

//rename image

periodindex = indexOf(OriNeuriteImage, ".");

if(periodindex >= 0) OriNeuriteImage = substring(OriNeuriteImage, 0, periodindex);

rename(OriNeuriteImage + "_" + "result");

//take measurements

print(" Analyzing " + OriNeuriteImage + "...");

setOption("BlackBackground", false);

run("Make Binary");

run("Set Measurements...", "area limit display redirect=None decimal=3");

run("Measure");

run("Analyze Particles...", "size=5-10000 circularity=0.20-1.00 show=[Overlay Masks] summarize");

//save images

selectWindow(OriNeuriteImage + "_" + "result");

run("Flatten");

saveAs("tiff", SaveDir + OriNeuriteImage + "_" + "result");

close(OriNeuriteImage + "_" + "result");

print(" Analysis of " + OriNeuriteImage + " complete");

}

IJ.renameResults("Summary", "Fragmented Neurite Area");

IJ.renameResults("Results", "Total Neurite Area");

selectWindow("Fragmented Neurite Area");

saveAs("Results", SaveDir +"Fragmented_Neurite_Area.csv");

selectWindow("Total Neurite Area");

saveAs("Results", SaveDir + "Total_Neurite_Area.csv");

print("Results have been saved in the selected directory.");

//save result

//saveAs("results", "C:\\Users\\kraemerb\\Desktop\\Dapi Test Folder\\Dir3")
