# Supplementary figures and images for: Quantification of Neurite Degeneration with Enhanced Accuracy and Efficiency in an In Vitro Model of Parkinson’s Disease
Source: eNeuro. 2022 Mar 17;9(2):ENEURO.0327-21.2022. doi: 10.1523/ENEURO.0327-21.2022 (PMC8938979; doi:10.1523/ENEURO.0327-21.2022)

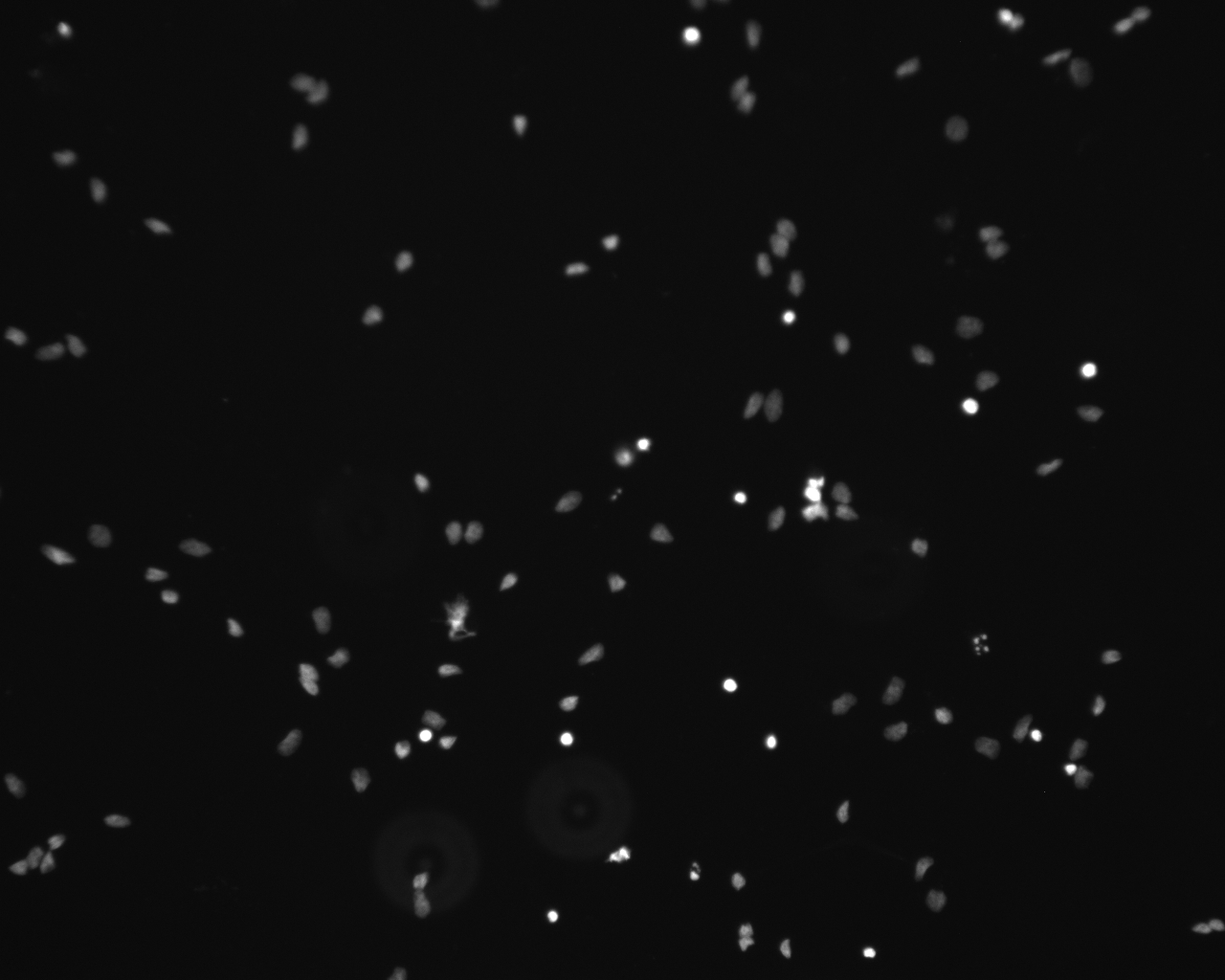

Supplement: Extended Data 1 — ANDI v1.1 script for image processing and DI analysis using ImageJ. Download Extended Data 1, ZIP file. [file enu-eN-MNT-0327-21-s02.zip › Example Image Set/DAPI/2_5_A.tif]

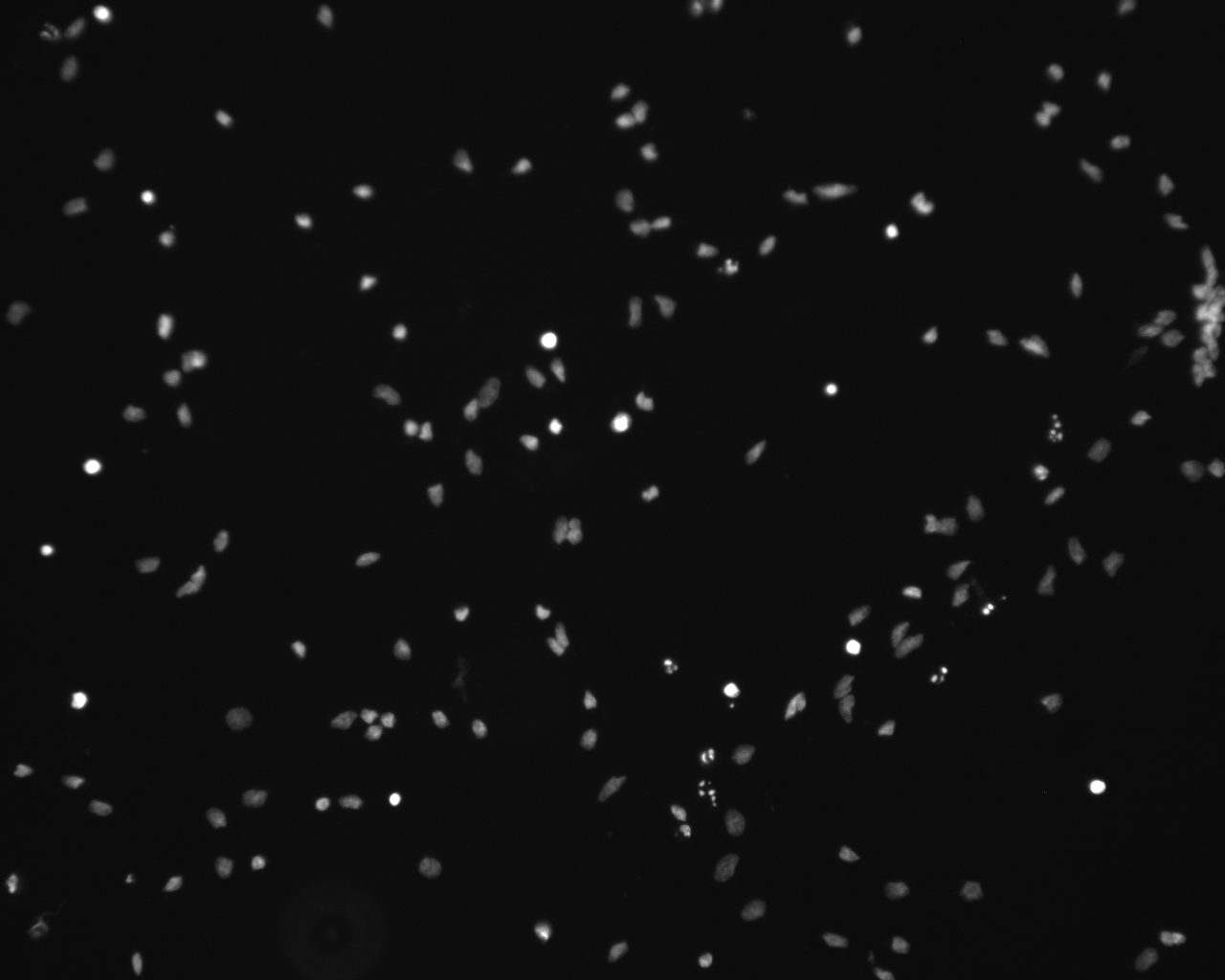

Supplement: Extended Data 1 — ANDI v1.1 script for image processing and DI analysis using ImageJ. Download Extended Data 1, ZIP file. [file enu-eN-MNT-0327-21-s02.zip › Example Image Set/DAPI/2_5_B.tif]

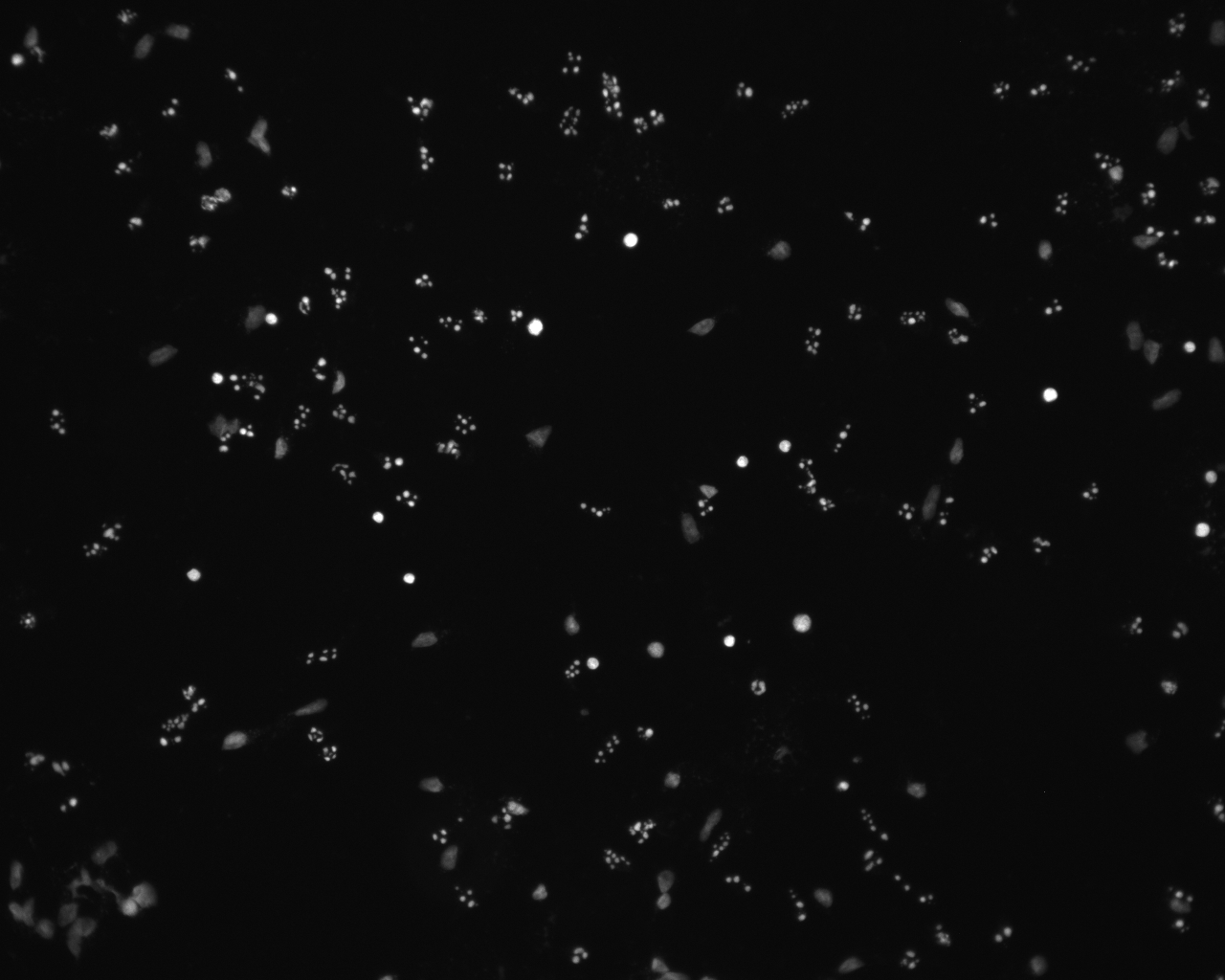

Supplement: Extended Data 1 — ANDI v1.1 script for image processing and DI analysis using ImageJ. Download Extended Data 1, ZIP file. [file enu-eN-MNT-0327-21-s02.zip › Example Image Set/DAPI/5_0_A.tif]

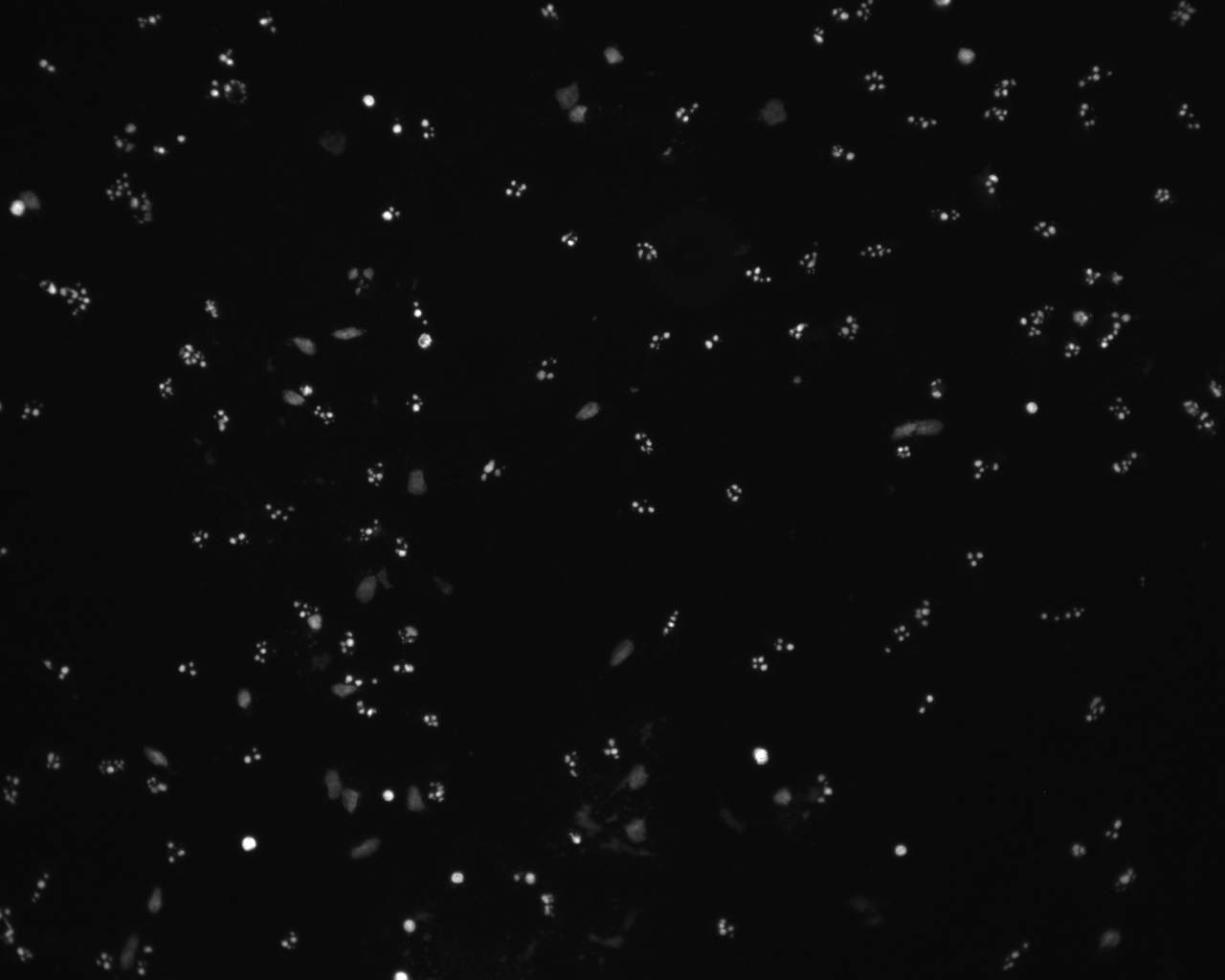

Supplement: Extended Data 1 — ANDI v1.1 script for image processing and DI analysis using ImageJ. Download Extended Data 1, ZIP file. [file enu-eN-MNT-0327-21-s02.zip › Example Image Set/DAPI/5_0_B.tif]

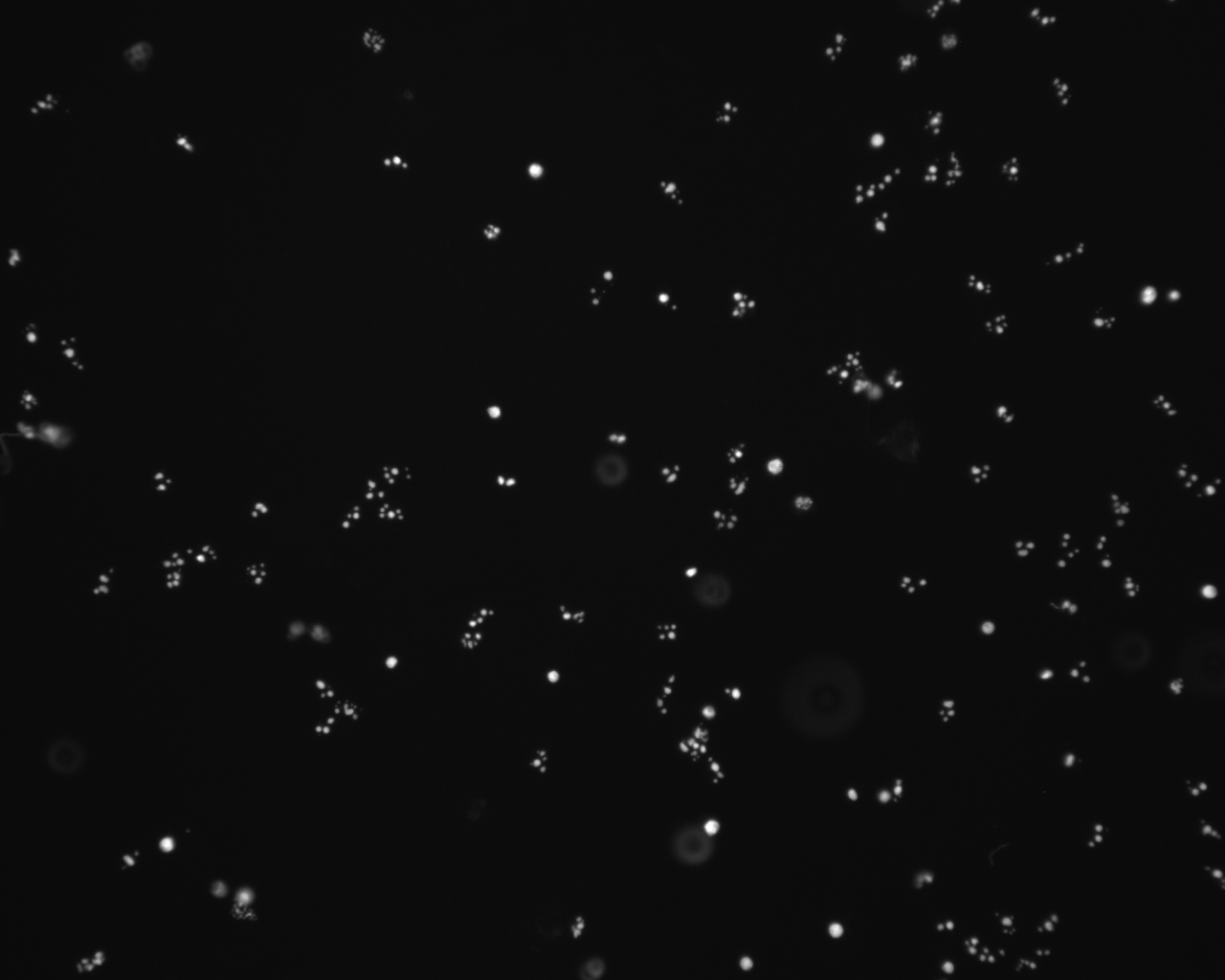

Supplement: Extended Data 1 — ANDI v1.1 script for image processing and DI analysis using ImageJ. Download Extended Data 1, ZIP file. [file enu-eN-MNT-0327-21-s02.zip › Example Image Set/DAPI/7_5_A.tif]

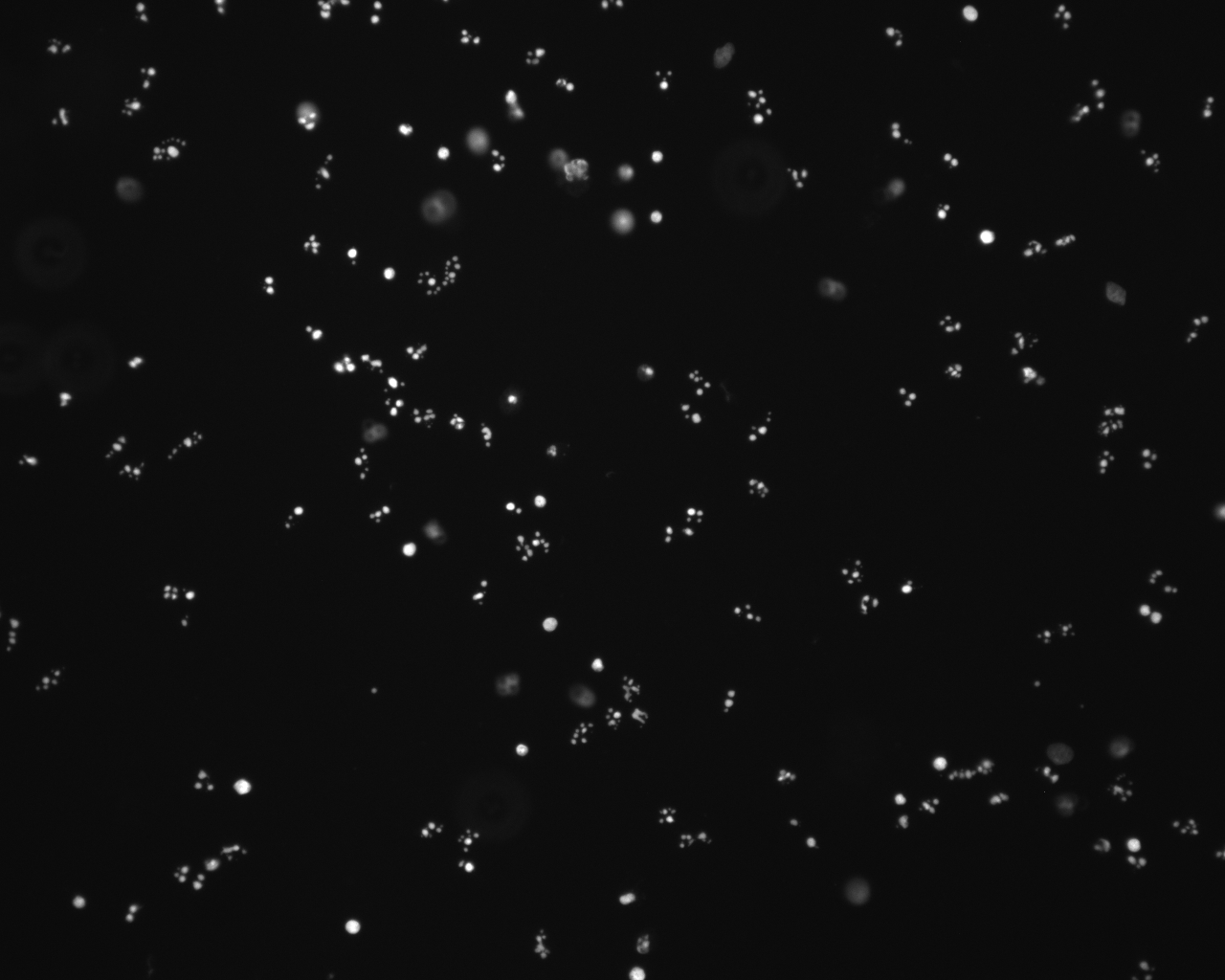

Supplement: Extended Data 1 — ANDI v1.1 script for image processing and DI analysis using ImageJ. Download Extended Data 1, ZIP file. [file enu-eN-MNT-0327-21-s02.zip › Example Image Set/DAPI/7_5_B.tif]

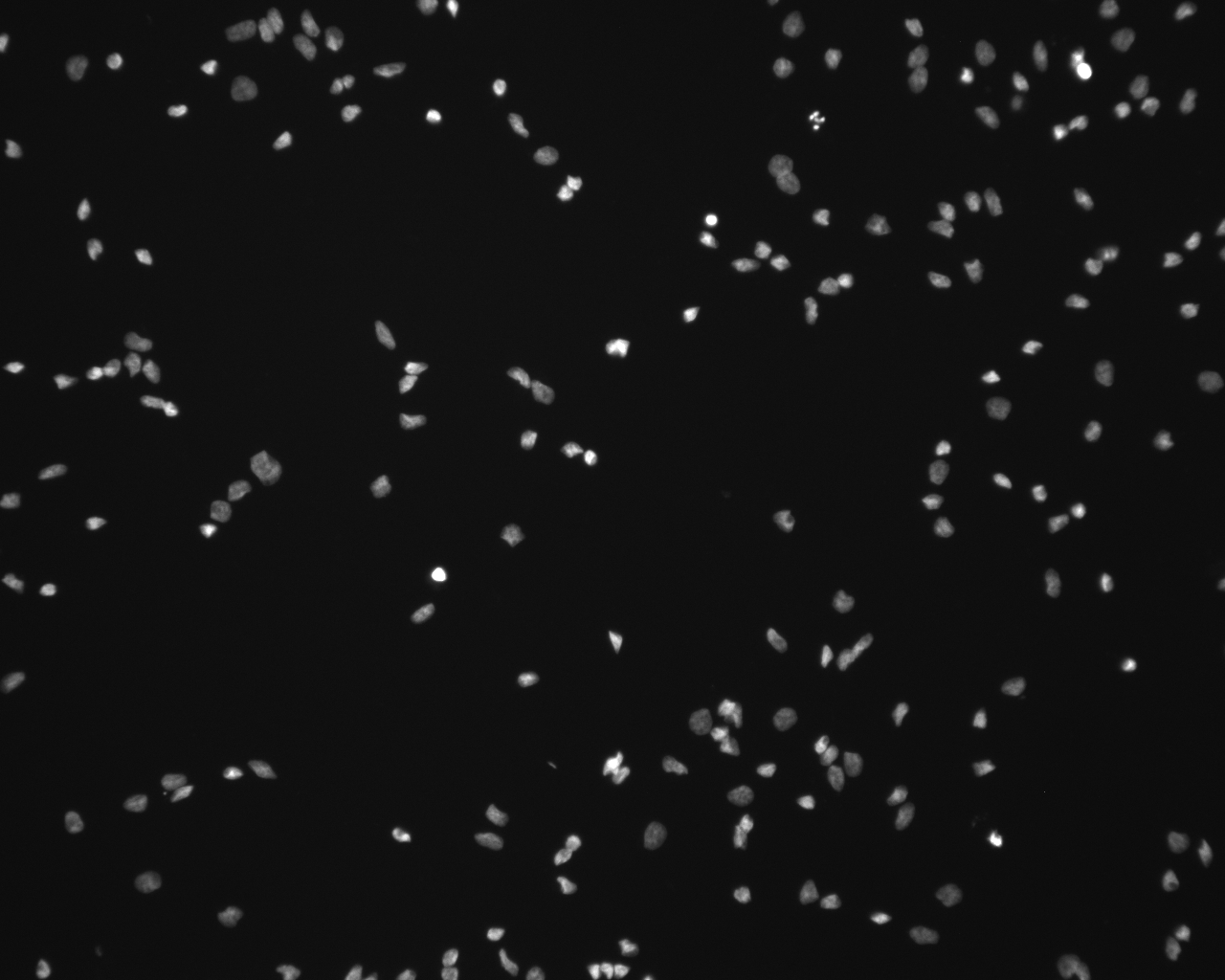

Supplement: Extended Data 1 — ANDI v1.1 script for image processing and DI analysis using ImageJ. Download Extended Data 1, ZIP file. [file enu-eN-MNT-0327-21-s02.zip › Example Image Set/DAPI/Veh_A.tif]

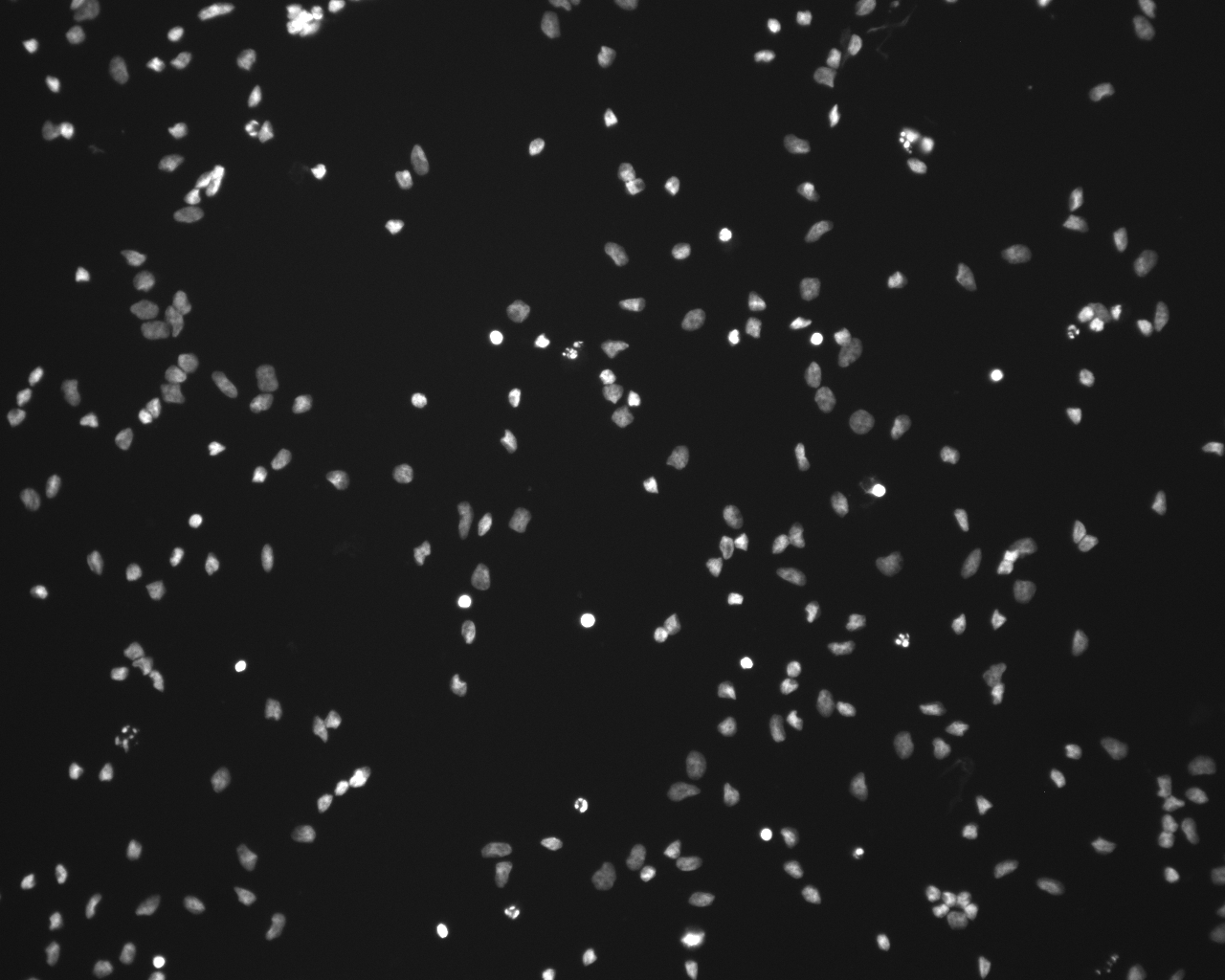

Supplement: Extended Data 1 — ANDI v1.1 script for image processing and DI analysis using ImageJ. Download Extended Data 1, ZIP file. [file enu-eN-MNT-0327-21-s02.zip › Example Image Set/DAPI/Veh_B.tif]

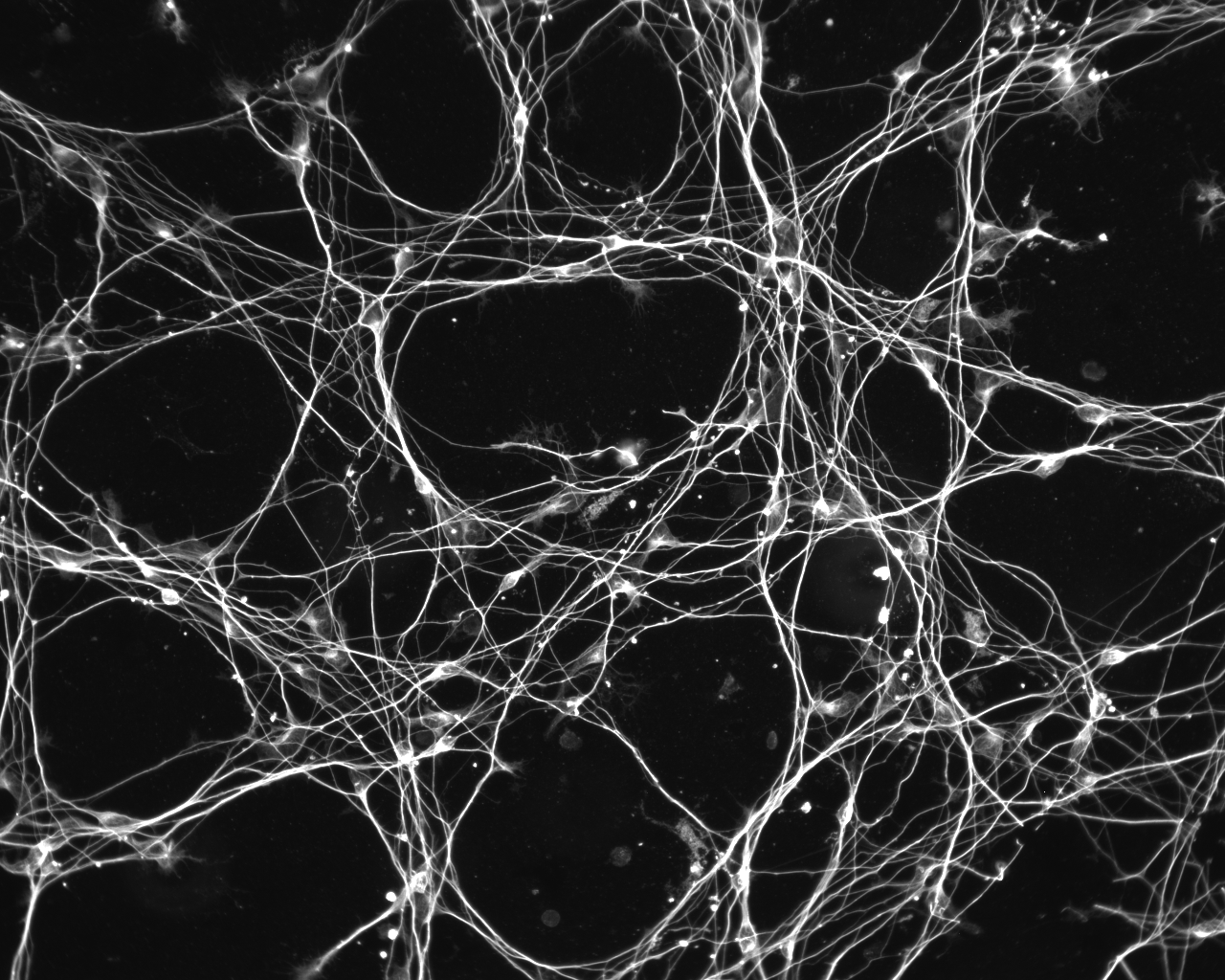

Supplement: Extended Data 1 — ANDI v1.1 script for image processing and DI analysis using ImageJ. Download Extended Data 1, ZIP file. [file enu-eN-MNT-0327-21-s02.zip › Example Image Set/Tubulin/2_5_A.tif]

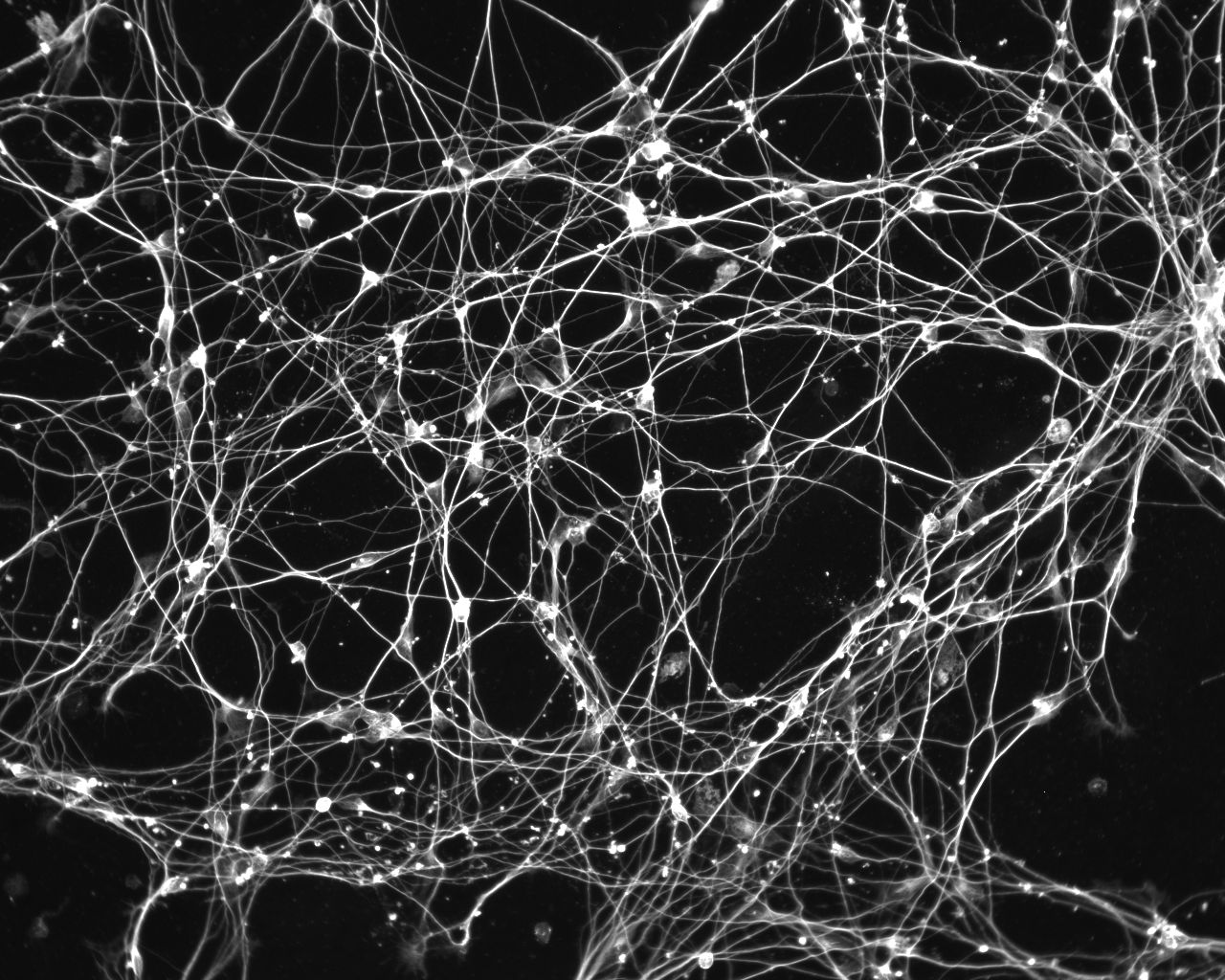

Supplement: Extended Data 1 — ANDI v1.1 script for image processing and DI analysis using ImageJ. Download Extended Data 1, ZIP file. [file enu-eN-MNT-0327-21-s02.zip › Example Image Set/Tubulin/2_5_B.tif]

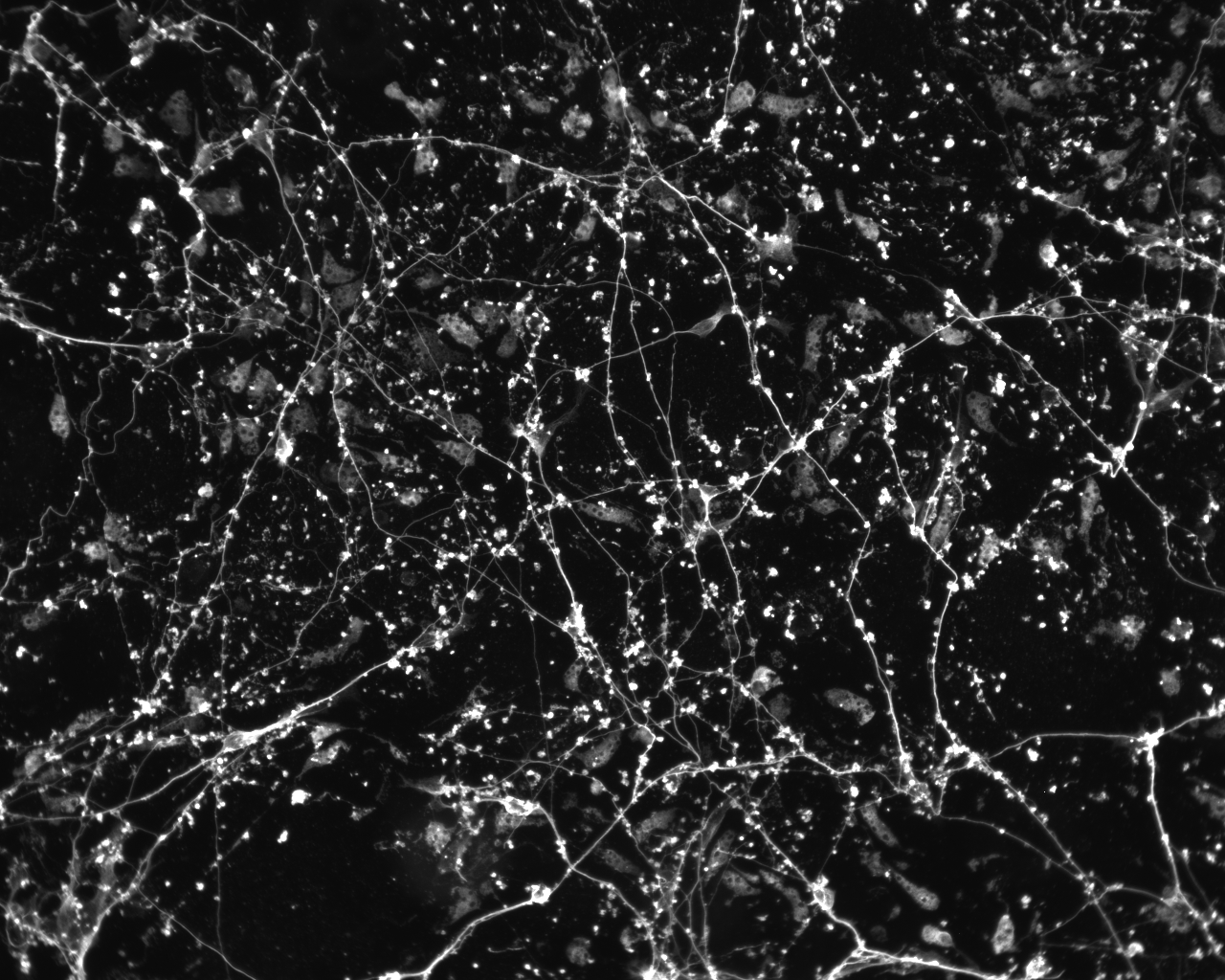

Supplement: Extended Data 1 — ANDI v1.1 script for image processing and DI analysis using ImageJ. Download Extended Data 1, ZIP file. [file enu-eN-MNT-0327-21-s02.zip › Example Image Set/Tubulin/5_0_A.tif]

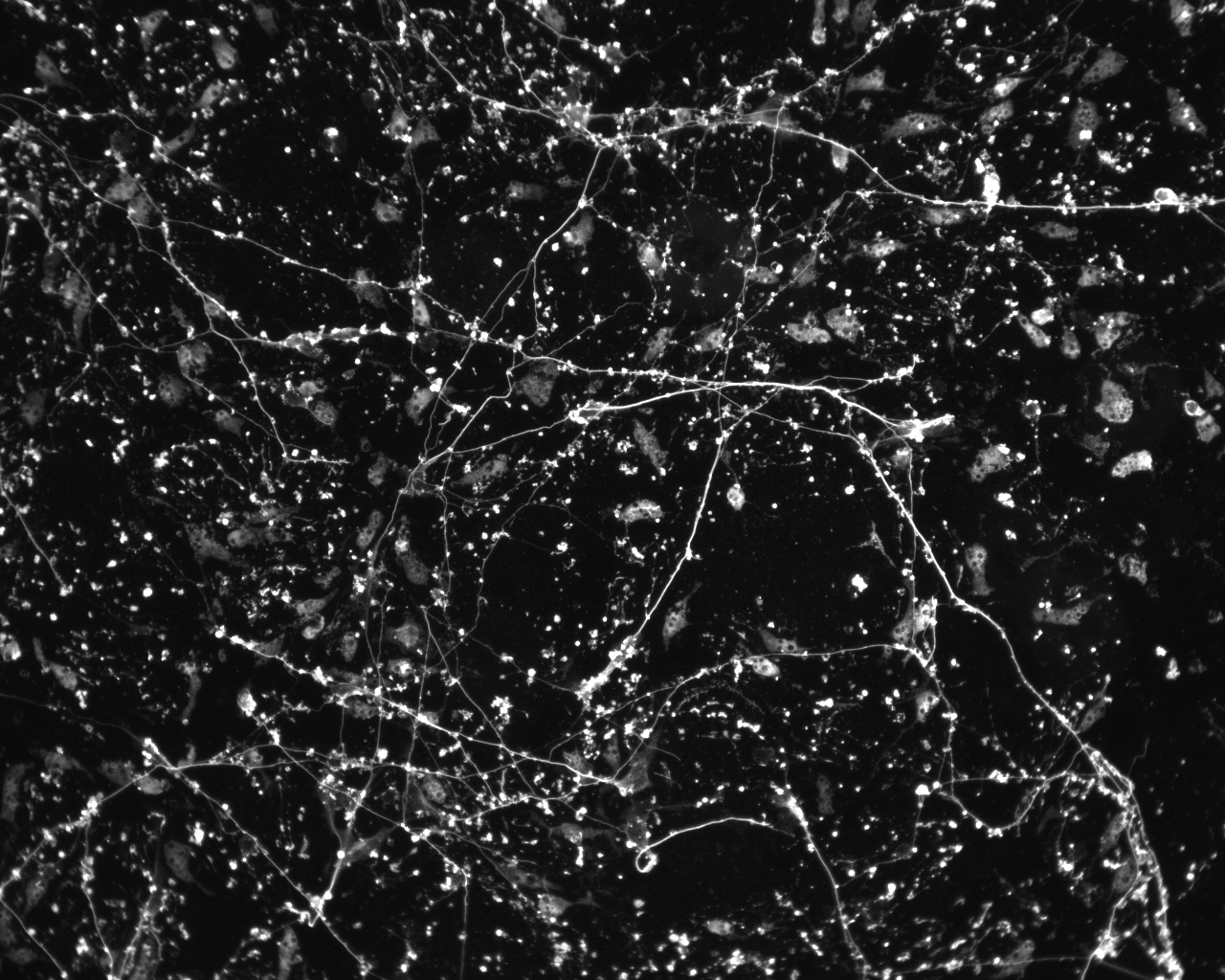

Supplement: Extended Data 1 — ANDI v1.1 script for image processing and DI analysis using ImageJ. Download Extended Data 1, ZIP file. [file enu-eN-MNT-0327-21-s02.zip › Example Image Set/Tubulin/5_0_B.tif]

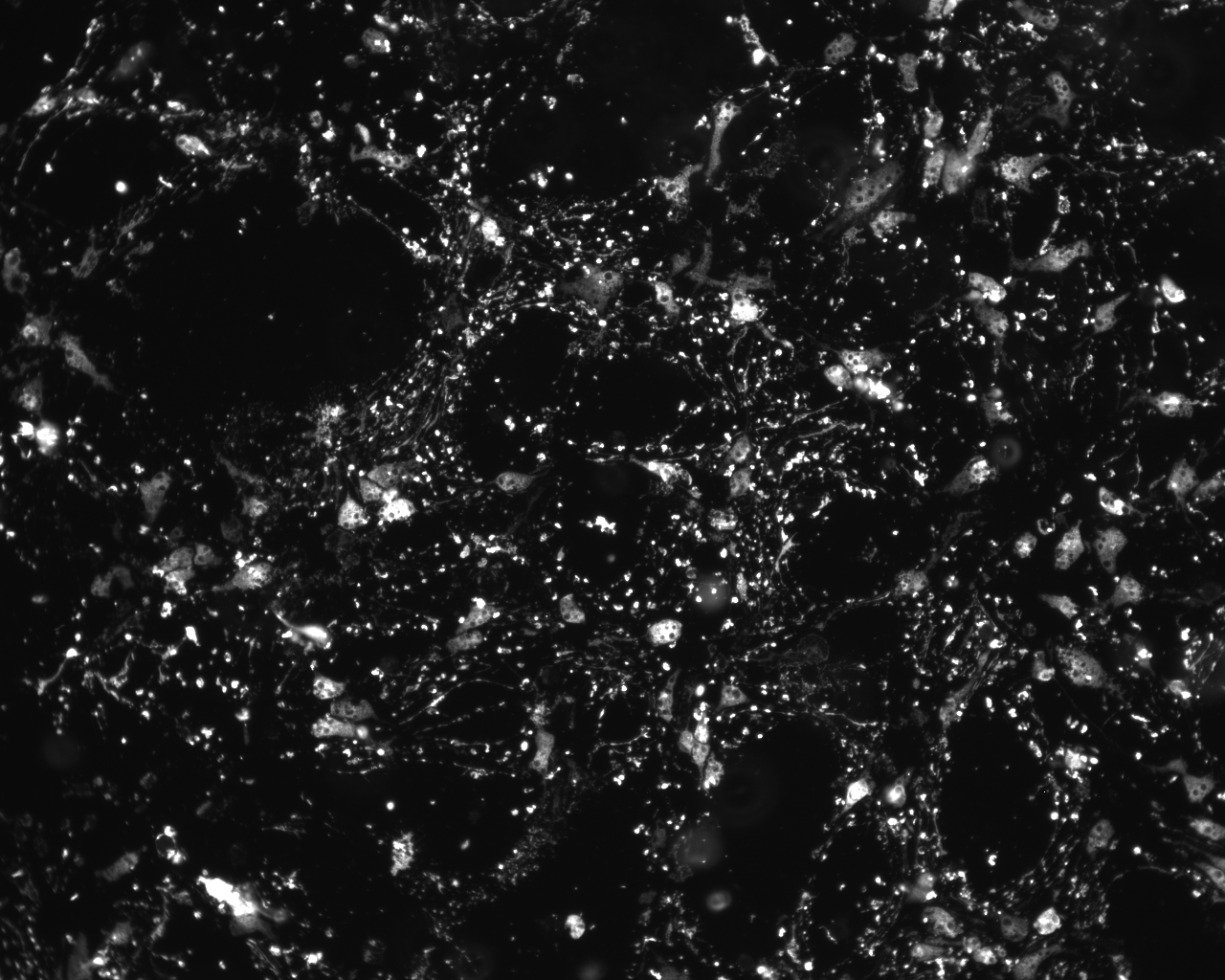

Supplement: Extended Data 1 — ANDI v1.1 script for image processing and DI analysis using ImageJ. Download Extended Data 1, ZIP file. [file enu-eN-MNT-0327-21-s02.zip › Example Image Set/Tubulin/7_5_A.tif]

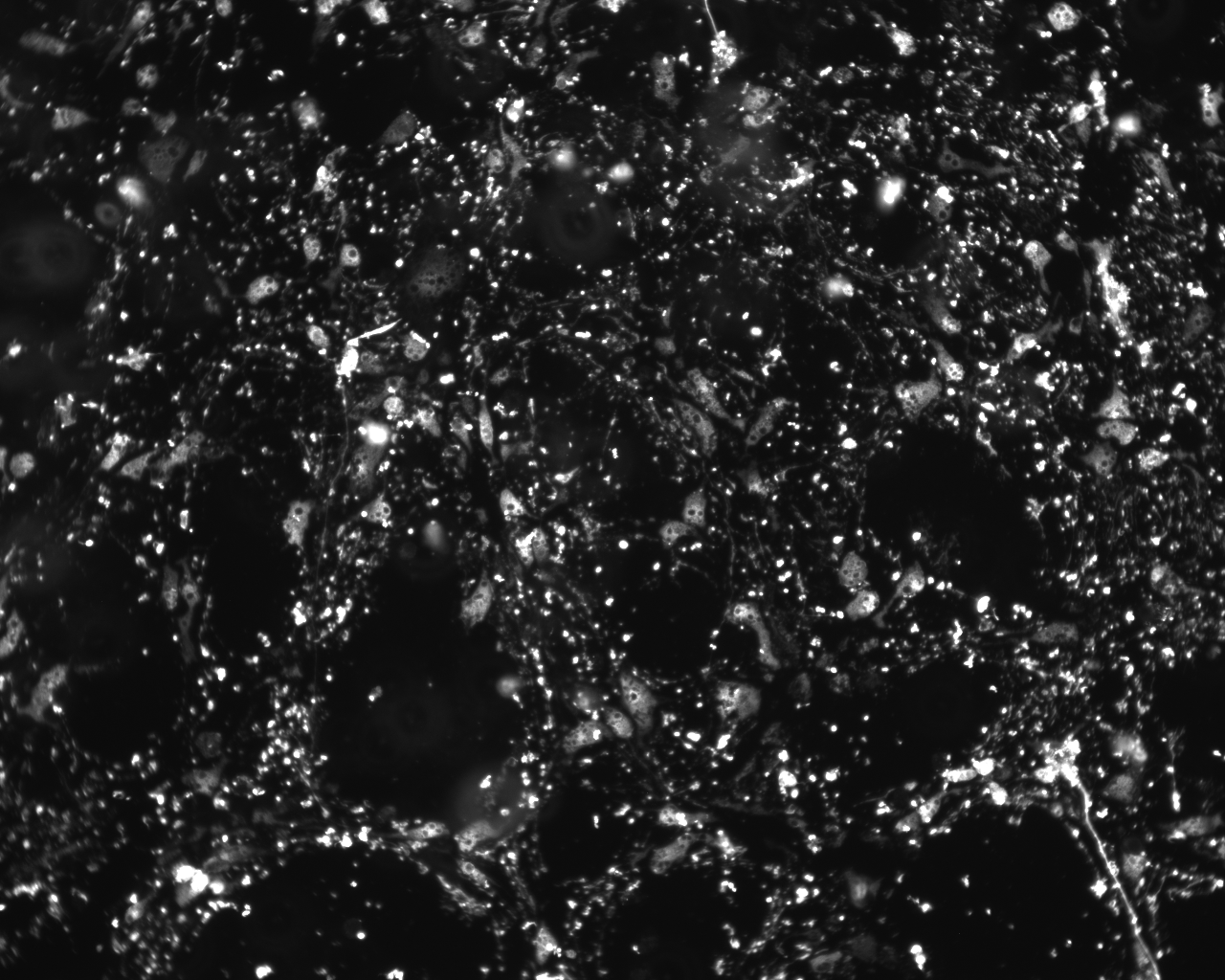

Supplement: Extended Data 1 — ANDI v1.1 script for image processing and DI analysis using ImageJ. Download Extended Data 1, ZIP file. [file enu-eN-MNT-0327-21-s02.zip › Example Image Set/Tubulin/7_5_B.tif]

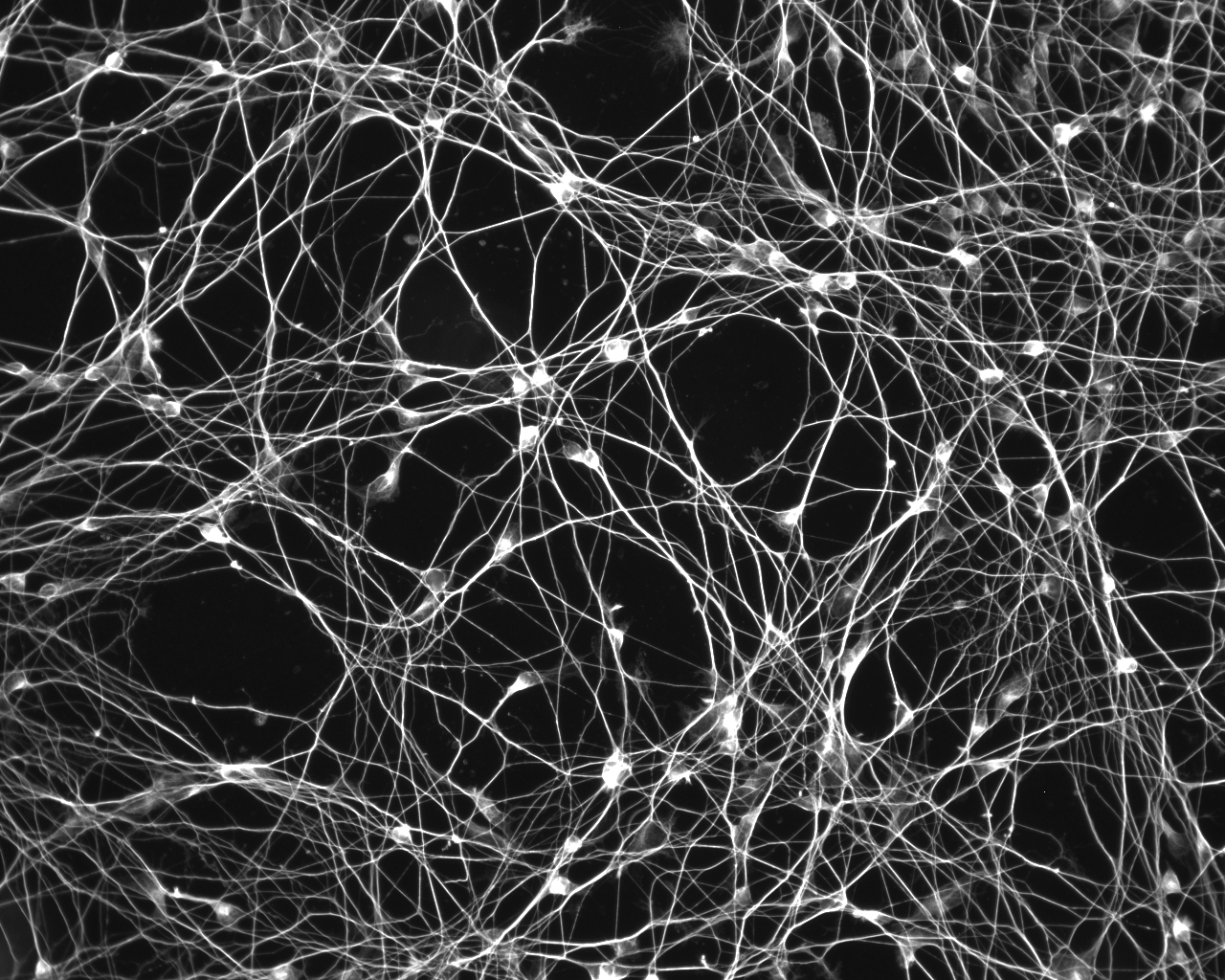

Supplement: Extended Data 1 — ANDI v1.1 script for image processing and DI analysis using ImageJ. Download Extended Data 1, ZIP file. [file enu-eN-MNT-0327-21-s02.zip › Example Image Set/Tubulin/Veh_A.tif]

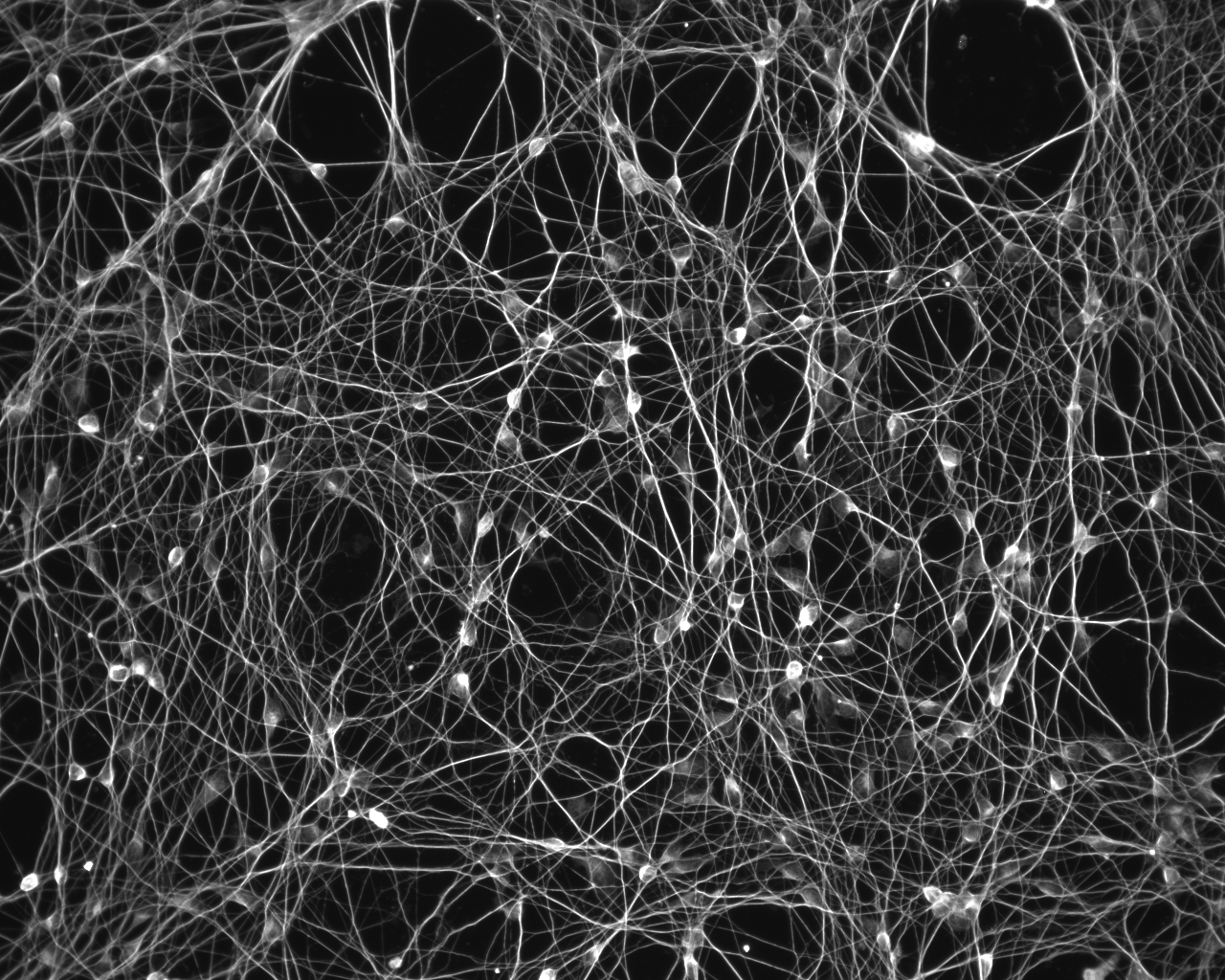

Supplement: Extended Data 1 — ANDI v1.1 script for image processing and DI analysis using ImageJ. Download Extended Data 1, ZIP file. [file enu-eN-MNT-0327-21-s02.zip › Example Image Set/Tubulin/Veh_B.tif]
